# Supplementary material for: SynBot is an open-source image analysis software for automated quantification of synapses
Source: Cell Rep Methods. 2024 Sep 9;4(9):100861. doi: 10.1016/j.crmeth.2024.100861 (PMC11440803; doi:10.1016/j.crmeth.2024.100861)
Supplement: Document S2. Article plus supplemental information [file mmc2.pdf]

Cell Reports
Methods

Article

SynBot is an open-source image analysis software for automated quantification of synapses

Graphical abstract

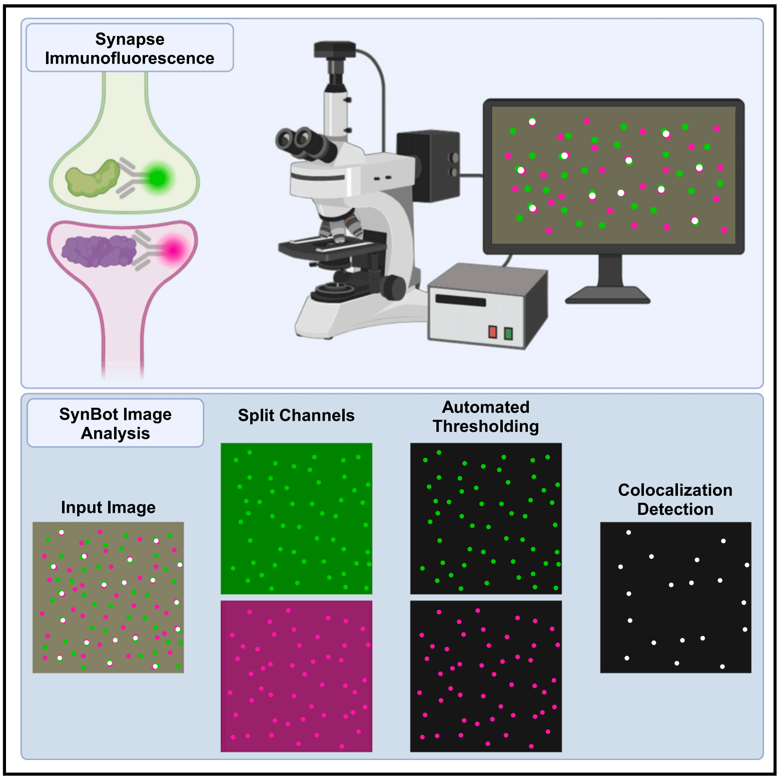

Highlights

- SynBot facilitates rapid analysis of synapses from immunofluorescence images
- SynBot includes ilastik and SynQuant algorithms to automatically threshold images
- SynBot accurately quantifies synapse numbers from *in vitro* or *in vivo* images
- SynBot is open source to enable widespread use and further development

Authors

Justin T. Savage, Juan J. Ramirez, W. Christopher Risher, Yizhi Wang, Dolores Irala, Cagla Eroglu

Correspondence

dolores.irala@duke.edu (D.I.),
cagla.eroglu@duke.edu (C.E.)

In brief

Immunofluorescence microscopy is a common approach for measuring synapse numbers. Savage et al. introduce and validate SynBot, an open-source software that automates the analysis of these images. This software provides a freely available platform with the potential to hasten discovery in the neuroscience field.

Savage et al., 2024, Cell Reports Methods *4*, 100861
September 16, 2024 © 2024 The Author(s). Published by Elsevier Inc.
https://doi.org/10.1016/j.crmeth.2024.100861

Cell Reports Methods

CellPress
OPEN ACCESS

Article

SynBot is an open-source image analysis software for automated quantification of synapses

Justin T. Savage,[1] Juan J. Ramirez,[1] W. Christopher Risher,[2] Yizhi Wang,[3] Dolores Irala,[4,*] and Cagla Eroglu[1,4,5,6,7,*]
[1]Department of Neurobiology, Duke University Medical Center, Durham, NC 27710, USA
[2]Department of Biomedical Sciences, Joan C. Edwards School of Medicine at Marshall University, Huntington, WV 25755, USA
[3]Bradley Department of Electrical and Computer Engineering, Virginia Polytechnic Institute and State University, Arlington, VA 22203, USA
[4]Department of Cell Biology, Duke University Medical Center, Durham, NC 27710, USA
[5]Howard Hughes Medical Institute, Duke University Medical Center, Durham, NC 27710, USA
[6]Aligning Science Across Parkinson's (ASAP) Collaborative Research Network, Chevy Chase, MD 20815, USA
[7]Lead contact
*Correspondence: dolores.irala@duke.edu (D.I.), cagla.eroglu@duke.edu (C.E.)
https://doi.org/10.1016/j.crmeth.2024.100861

MOTIVATION Light microscopy imaging of pre- and post-synaptic proteins from neurons in tissue or *in vitro* allows for the effective identification of synaptic structures. Previous methods for quantitative analysis of these images were time consuming and required extensive user training, and the source code could not be easily modified. Here, we describe SynBot, an open-source tool that automates the synapse quantification process, decreases the requirement for user training, and allows for easy modifications to the code.

SUMMARY

The formation of precise numbers of neuronal connections, known as synapses, is crucial for brain function. Therefore, synaptogenesis mechanisms have been one of the main focuses of neuroscience. Immunohistochemistry is a common tool for visualizing synapses. Thus, quantifying the numbers of synapses from light microscopy images enables screening the impacts of experimental manipulations on synapse development. Despite its utility, this approach is paired with low-throughput analysis methods that are challenging to learn, and the results are variable between experimenters, especially when analyzing noisy images of brain tissue. We developed an open-source ImageJ-based software, SynBot, to address these technical bottlenecks by automating the analysis. SynBot incorporates the advanced algorithms ilastik and SynQuant for accurate thresholding for synaptic puncta identification, and the code can easily be modified by users. The use of this software will allow for rapid and reproducible screening of synaptic phenotypes in healthy and diseased nervous systems.

INTRODUCTION

Proper brain function relies on the correct wiring of neuronal networks.[1–4] Asymmetric neuron-neuron connections, synapses, are the fundamental biological units of neural circuits.[5–7] Importantly, synapse loss or dysfunction is a hallmark of many neurodevelopmental and neurodegenerative disorders.[8–10] Therefore, studying synapse connectivity is crucial to understanding how neuronal circuits are established during development, remodeled throughout life, and impacted by diseases.

Synapses are composed of two neuronal structures: the pre-synapse, located in the axon terminal, and the post-synapse, located on the dendrite. The pre-synapse contains specialized, neurotransmitter-filled synaptic vesicles.[6,11] Vesicles are docked at the pre-synaptic active zone and fused with the membrane when an action potential reaches the synapse.[11] Neurotransmitters then diffuse into the extracellular space between the pre- and post-synapses, called the synaptic cleft.[12] Neurotransmitters in the synaptic cleft bind post-synaptic neurotransmitter receptors.[13–15] These receptors are transmembrane proteins that pass ions and/or recruit intracellular signaling partners to transduce a signal into the post-synaptic cell[7,13–15] and are anchored at the post-synapse through a scaffold of post-synaptic density proteins.[16,17]

Synapses are dynamic structures that can be strengthened or lost due to changes in the inputs that neurons receive.[18–20] Conversely, perturbations in genes controlling synaptogenesis also impact the number and organization of synaptic structures.[21–23] Non-neuronal cell types, such as astrocytes, microglia, and oligodendrocytes, also serve as critical regulators of synapse formation and elimination.[24–28] In particular, astrocytes strongly induce the formation of excitatory and inhibitory

This is an open access article under the CC BY license (http://creativecommons.org/licenses/by/4.0/).

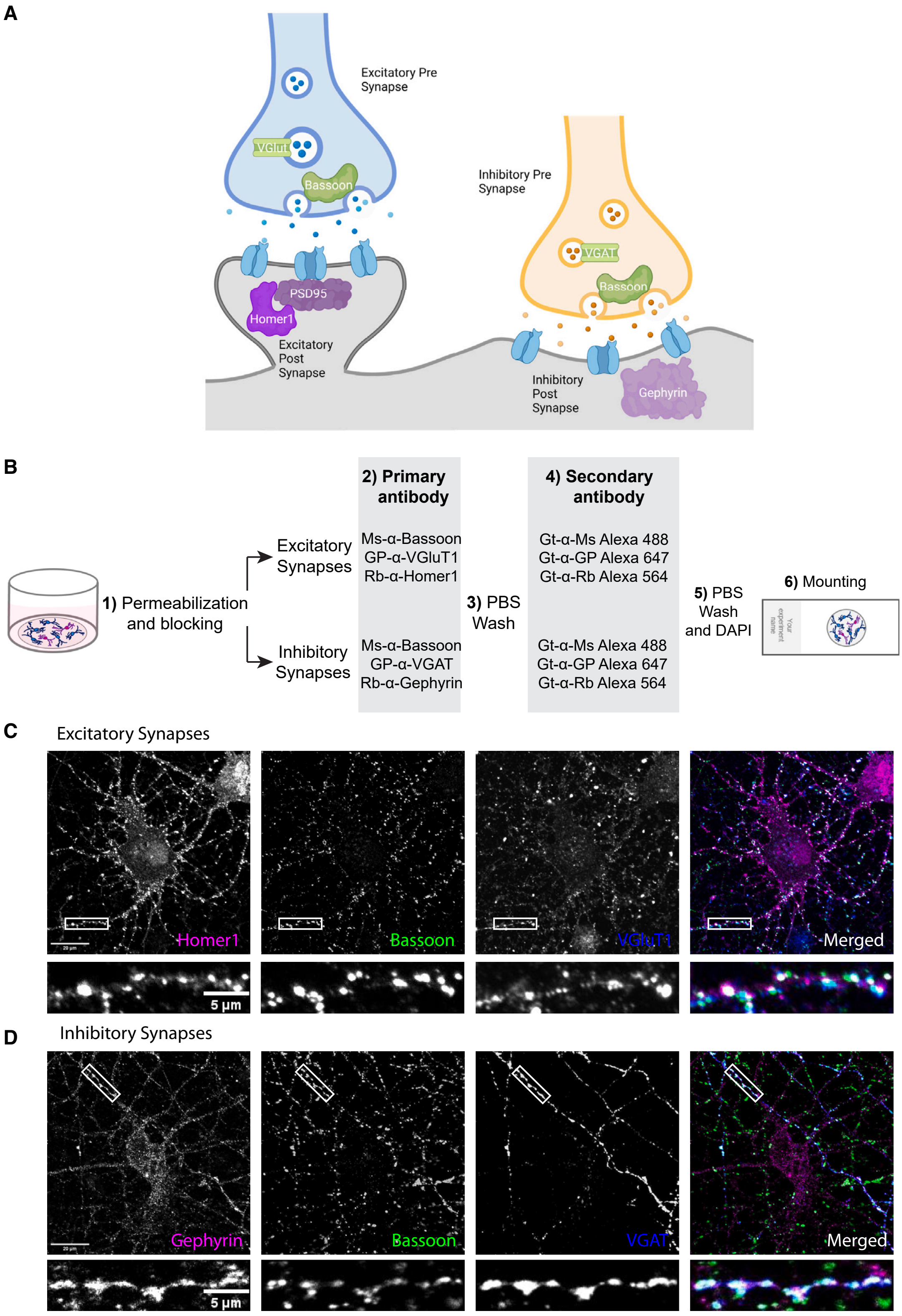

(legend on next page)

synapses through direct contact with neuronal processes or via the secretion of several synaptogenic proteins (reviewed in Farhy-Tselnicker and Allen,[1] Allen and Eroglu,[29] Baldwin and Eroglu,[30] and Tan et al.[31]).

The gold-standard methods for interrogating the structure and function of synapses are electron microscopy (EM) and electrophysiology, respectively. EM allows the experimenter to visualize the synapse with a high enough resolution (~2 nm) to resolve the pre- and post-synaptic compartments individually.[32–34] The characteristics of pre-synaptic vesicles and post-synaptic densities enable the identification of excitatory versus inhibitory synapses.[34–36] Analyzing the number of opposing pre- and post-synaptic sites in electron micrographs of samples from various conditions can determine if these conditions alter the number of synaptic structures.[34–36]

A common method for investigating synaptic function is the whole-cell patch-clamp analysis of miniature post-synaptic currents. The frequency of miniature post-synaptic currents provides information about the number of synapses a cell receives or the probability of release at the pre-synaptic site. Additionally, the amplitude of these currents measures post-synaptic strength.[37]

EM and electrophysiology continue to provide high-resolution structural and functional information about synaptic connectivity, but these techniques have major limitations. First, they require extensive sample preparation and specialized equipment, making them difficult to establish in a new laboratory. Second, they have very low throughput and sample only a small subset of synapses or neurons, making them unsuited for screening multiple experimental conditions.

To address these limitations, higher-throughput methods for assessing synapse numbers using immunostaining have proven useful.[38,39] These histological methods use antibodies to label pairs of pre- and post-synaptic proteins that are specialized to distinct synaptic subtypes. For example, pre-synaptic markers, such as vesicular glutamate transporter 1 (VGluT1), VGluT2, or Bassoon, can be paired with the post-synaptic markers post-synaptic density protein 95 (PSD95) or Homer-1 to label excitatory synapses.[40–44] Similarly, the vesicular GABA transporter (VGAT), together with the post-synaptic gephyrin, mark inhibitory synapses.[41,43,45] See Verstraelen et al. for a detailed comparison of synaptic marker performances.[46] When these markers are imaged using light microscopy, due to the resolution limit (200–300 nm)[47] and the short (20–30 nm)[44,48] distance between the pre- and post-synaptic compartments, the color signals appear to overlap at synapses.

Therefore, synaptic structures can be quantified by counting the number of colocalized pre- and post-synaptic markers using specialized image analysis software. One of the first programs for performing this analysis is the Puncta Analyzer plugin for ImageJ,[38] which has been widely used in neuroscience and yielded results that were then validated through EM and electrophysiology.[41,45,49,50]

A major limitation of the Puncta Analyzer is that all analysis steps require user input, which is time consuming and can be highly subjective. Thus, image analysis using the Puncta Analyzer is a lengthy process requiring extensive user training. In addition, the source code for the Puncta Analyzer is complex and difficult to edit to allow for customization.

To circumvent these technical challenges, several alternative analysis pipelines have been developed that automate portions of synapse counting. These include methods to count synapses along a neurite (SynD and SynPAnal),[51,52] using additional filtering to improve manual thresholding (SynapseJ[53]), using automated thresholding algorithms in FIJI (Synapse Counter),[54] or development of statistical thresholding algorithms (SynQuant).[55] While these methods provided important features beyond what is available in the Puncta Analyzer, none have been as widely used as the Puncta Analyzer, which is well suited to quantify densely packed synapses like those seen in the mouse brain tissues. To circumvent the limitations of the Puncta Analyzer, here we developed and tested an open-source ImageJ-based synapse analysis software called SynBot, which is optimized for not only *in vitro* but also high-noise *in vivo* images and allows for a wide variety of options to tailor the analysis to the experimenter's needs. We compared the accuracy and efficiency of SynBot with the Puncta Analyzer using simulated and experimental data, which were previously validated by EM and electrophysiology.[50,56]

RESULTS

Synapse labeling by immunohistochemistry

Synapse quantification relies on the use of immunohistochemistry to fluorescently label the pre- and post-synaptic compartments (Figure 1A). A standard immunostaining workflow includes (1) fixation and permeabilization, (2) application of primary antibodies against pre-synaptic and post-synaptic markers, (3) application of fluorescent secondary antibodies, and (4) image acquisition by fluorescence microscopy (Figure 1B). This protocol results in images where either excitatory (Figure 1C) or inhibitory (Figure 1D) synapses can be visualized.

Figure 1. Labeling of synapses as apposing colocalizations of pre- and post-synaptic markers

(A) Illustration of excitatory and inhibitory synaptic structures. Pre-synaptic markers used in this paper are shown in green, and post-synaptic markers are shown in magenta.

(B) Synaptic staining workflow. (1) After fixation, neurons are permeabilized, and non-specific epitopes are blocked in antibody buffer. (2) Neurons are incubated with primary antibodies targeting pre- and post-synaptic compartments of excitatory or inhibitory synapses. (3) After overnight incubation with primary antibodies, the neurons are washed with PBS. (4) Neurons are incubated with secondary antibodies conjugated to Alexa fluorophores. (5) After 2 h of room temperature incubation with secondary antibodies, neurons are again washed with PBS, and the neuronal nuclei are stained with DAPI. (6) Coverslips are mounted on glass slides using a medium that protects from bleaching of Alexa fluorophores for imaging. For details, check the STAR Methods section and protocols.io.

(C) Representative image of excitatory synapses made onto a cultured cortical neuron stained with Homer1, Bassoon, and VGluT1. The scale bar in the larger image represents 20 μm, and the scale bar in the smaller image represents 5 μm.

(D) Representative image of inhibitory synapses made onto a cultured cortical neuron stained with Gephyrin, Bassoon, and VGAT. The scale bar in the larger image represents 20 μm, and the scale bar in the smaller image represents 5 μm.

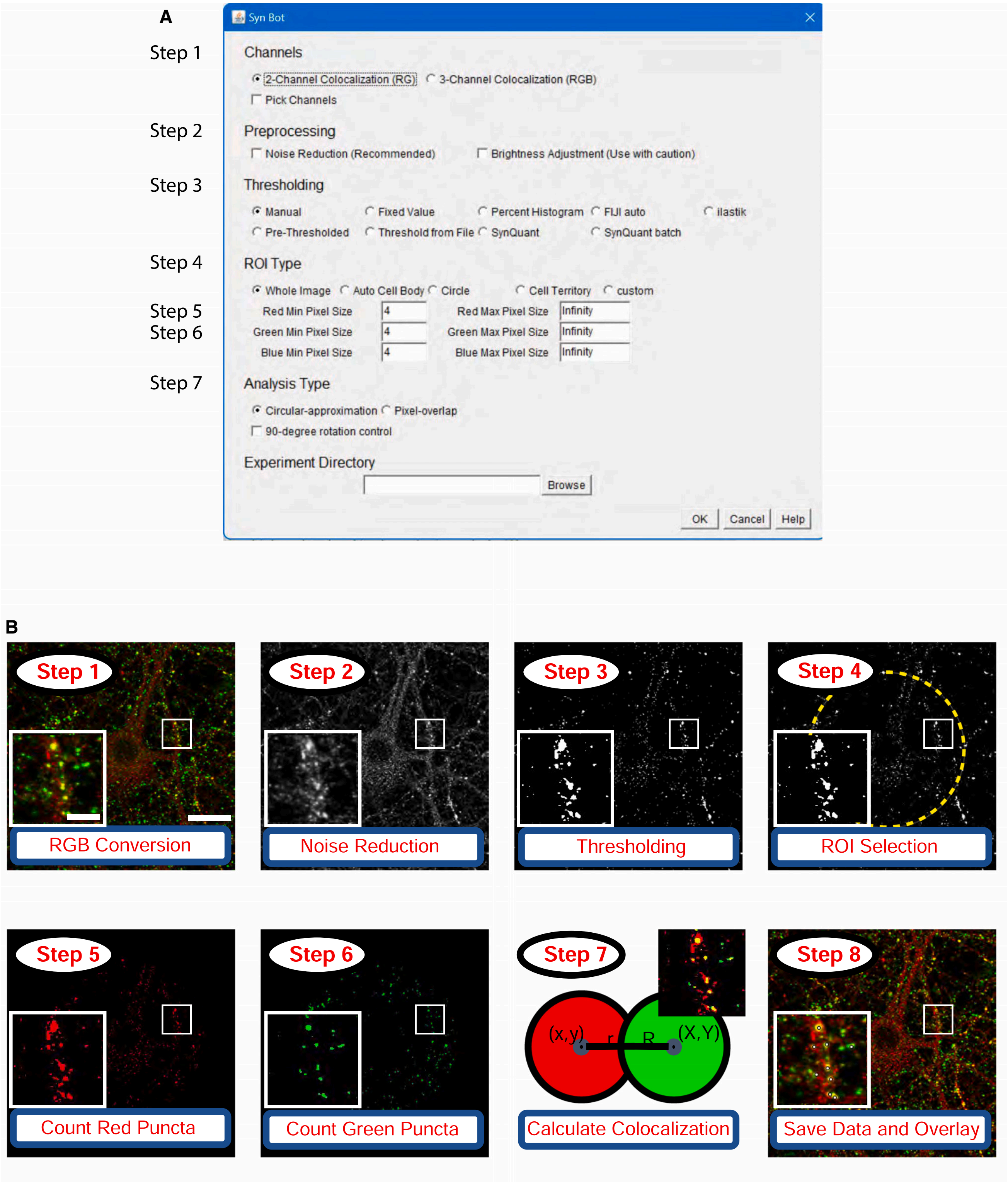

Figure 2. Workflow for SynBot synapse quantification

(A) The SynBot selection menu serves as the primary point of user input to adjust options for synapse analysis.

(B) The main steps in the SynBot colocalization analysis. (1) Images are loaded into the software and converted to RGB. (2) Images are denoised using the FIJI subtract background and Gaussian blur filter functions. (3) Each image channel is thresholded to separate synaptic puncta from the background. (4) A region of

(legend continued on next page)

CellPress
OPEN ACCESS

The details of the methods are explained in the STAR Methods section and at protocols.io (https://www.protocols.io/view/synbot-protocols-3byl4qewjvo5/v2).

Developing an automated synapse quantification tool

Our first goal in developing SynBot was to enable the efficient quantification of large imaging datasets. SynBot combines many of the image processing and analysis steps that were performed for analysis with the Puncta Analyzer into one automated workflow. We developed an ImageJ macro to automate the pre-processing steps that convert raw images into numerical representations used to calculate synaptic colocalizations (Figure 2). First, SynBot determines if input images are z stacks of confocal images or single images. Then, SynBot processes the z stack files to produce max projections of each 1 μm stack. This step is of particular use for *in vivo* synapse number analyses, which will be discussed in detail in a later section. Second, all images are converted to the RGB format, the color format ImageJ uses. Third, based on user selection, the FIJI subtract background and Gaussian blur filter functions are applied to remove noise from the image. The fourth step is the thresholding of individual synaptic puncta to set a value for the background of the image and exclude the pixels in the image that have an intensity value less than the background. A threshold value can be determined in several different ways, which are discussed in the next section. Fifth, the user decides whether to quantify synapses within a region of interest (ROI) or the whole image. After entering these user inputs, the program runs the FIJI analyze particles function to record the location and area of each punctum in the ROI of the thresholded image. This function records the x and y coordinates and area of each punctum for each color channel. Each punctum is then compared to the puncta in the other channel of the image by approximating each punctum to a circle or through a pixel-by-pixel approach, discussed below.

SynBot analysis workflow

The files required for running SynBot and the installation instructions can be found at https://github.com/Eroglu-Lab/Syn_Bot. There are also tutorials and instructional videos at https://www.protocols.io/view/synbot-protocols-3byl4qewjvo5/v2. Once the Syn_Bot.ijm macro is started, a menu allows the user to select their analysis parameters (Figure 2). The menu has 6 main sections: (1) channels, (2) pre-processing, (3) thresholding, (4) ROI type, (5) analysis type, and (6) experiment directory. The detailed functions of each option on the SynBot selection menu are defined in Table S1. Below, we summarize the utility of each section.

(1) Channels: the fundamental objective of SynBot is to determine the overlap between different color channel objects (i.e., pre- or post-synaptic puncta). SynBot does this by first converting images into the red, green, and blue (RGB) format, which allows for the consistent labeling of channels throughout the program. Users can select either the 2-channel option to analyze colocalization between the red and green channels or the 3-channel option to analyze colocalization between the RGB channels. If your images are in CMYK (cyan, magenta, yellow, and key [black]) format, or you would like to analyze red and blue or green and blue puncta colocalization, then there is also the "pick channels" option. z stack images are max projected to produce individual projections that SynBot analyzes as separate images. For the experiments described below, every 3 z stacks were combined to produce a 1 μm max projection. The number of stacks to project together can be adjusted by the user or set to 1 to allow individual processing of each optical section.

(2) Pre-processing: images can be pre-processed after RGB conversion to reduce noise or adjust the brightness. Noise reduction is performed by applying FIJI's subtract background plugin followed by a Gaussian blur filter to aid in object detection. Brightness adjustment changes the intensity values of each image such that there is an equal percentage of saturated pixels across images. As these methods modify the image data that SynBot will use for analysis, we advise users to apply them with caution and ensure they are producing accurate results. These modifications should also be consistent across groups of images that will be included in the same experiment.

(3) Thresholding: one of the most challenging aspects of image analysis is thresholding, the process of distinguishing the foreground (i.e., synaptic puncta) of an image from the background. Discriminating between true synaptic puncta and the background noise is not trivial due to the small size of the synaptic puncta and the variability in staining and imaging parameters between users and microscopes. Therefore, here we include a number of possible options that SynBot users can utilize. First, there is manual thresholding, where the user selects a threshold value for each channel of each image and SynBot runs the rest of the analysis. This option is similar to the Puncta Analyzer, the predecessor of SynBot. However, we designed SynBot to have several time-saving, user-friendly features. The manual thresholding method requires extensive user training to use properly and necessitates a significant amount of hands-on user time.

To address the limitations of manual thresholding, we implemented several automated thresholding methods. First, we created an ilastik-based[57] thresholding method, where a machine learning model is used to threshold each channel of each image. ilastik is an open-source program trained by the user in a small number of images (typically 3–5) to threshold by extracting a set of user-defined features from the image. ilastik then feeds these features into a random forest machine learning model. This model is then applied to the rest of the images in the dataset to determine if a given pixel is

interest (ROI) for colocalization quantification can be selected (yellow dashed line). (5 and 6) Red and green puncta are detected by FIJI's analyze particles function and counted. (7) Colocalizations between red and green puncta are calculated. (8) The colocalized puncta are overlayed on the original image, and the data are saved as a CSV file. The scale bar in the larger image represents 20 μm, and the scale bar in the smaller image represents 5 μm.
See also Table S2.

part of the foreground or background of the image. As in all thresholding methods, ilastik requires careful troubleshooting to ensure accurate results. The training should be done for each experiment independently and should not be used across datasets unless they are collected under identical conditions, such as replicates of the same experiment performed by the same investigator. However, using ilastik has a distinct advantage: it can be applied to a large number of images without the need for further user input and only takes 5–15 min to generate the trained model.

In addition to ilastik, we also integrated the SynQuant algorithm for thresholding into SynBot. This method was developed by Wang et al. in 2020[55] to specifically threshold synapses from immunofluorescence images through a statistical-probability-based approach. This is explained in detail in Wang et al., but in short, the method assesses each potential synaptic object by comparing it to its neighboring pixels based on fluorescence intensity, object size, and local contrast. The SynQuant implementation within SynBot allows the user to adjust the following parameters: *Z* score threshold (a statistical measurement of how different values are from the mean), minimum object size, maximum object size, minimum object fill, maximum width-to-height ratio, and estimated noise standard deviation. For most datasets, the noise standard deviation and *Z* score threshold are the only parameters that require adjustment. The SynQuant batch processing of images through SynBot takes approximately 5–10 min for the size of datasets presented here without any need for user input.

We added another feature to Synbot to aid the reproducibility of results between users. All the thresholding values are saved for each image analyzed. Therefore, an analysis performed with SynBot can be efficiently reproduced later using the "threshold from file" option. This option does not work for the ilastik or SynQuant thresholding options because these algorithms do not use a set thresholding value, but they can already be easily reproduced since they are unsupervised methods.

(4) ROI type: SynBot's colocalization analysis can be targeted to an ROI within the input image. One common application is the restriction of the analysis to the region around the soma of a cultured neuron, as shown in Figure 5. Alternatively, users can implement their own complex ROIs by using the FIJI clear function to remove unwanted regions prior to running SynBot on the entire image. During the selection of the ROI, the user is also asked to input a minimum and a maximum pixel size for each channel puncta. The area of the ROI selected for each image is recorded and included in the output file.

(5) Analysis type: the Puncta Analyzer calculated the colocalization based on a circular approximation colocalization approach[38] (Figure 3). Therefore, we included this option also in SynBot. With the circular approximation analysis mode selected, the coordinates and radius of each punctum for the channels to be analyzed are compared to each punctum in the second channel to detect colocalizations and calculate their area using the geometry summarized in Figure 3. First, the distance between the centers of the two circles is calculated (Figure 3A). If this distance is less than the sum of the radii of the two circles, then they are counted as a colocalization, and the area of the colocalization is calculated (Figure 3B). The basic idea of the area calculation is to use the two intersection points of the circles along with the center of each circle to define two triangles while also finding the areas of the sector of each circle between the two intersection points. Subtracting the area of each triangle from the area of its surrounding sector gives half of the overlapping area contributed by that punctum. This can be done for both puncta to give the total area of colocalization.[58] The coordinates and area of each colocalization are then stored and added to the colocalized puncta count. This method only works if synaptic puncta have high circularity, which can be determined using the measure tool in FIJI. In our experience, most synaptic markers used in primary culture or the mouse cortex are approximately circular. A table of experimental circularity values for the antibodies used in this paper is provided in Table S2. We also directly compared this circular approximation to the pixel-by-pixel alternative and found nearly identical results for the types of images shown here (Figure S1).

The circular approximation method is too slow to practically analyze 3-channel images and fails to correctly calculate colocalizations between non-circular objects. To overcome these limitations, here we developed a pixel overlap colocalization method within SynBot (Figure 3C). Rather than approximating each punctum to a circle, this analysis mode compares puncta in a pixel-by-pixel fashion using the FIJI image calculator plugin's AND function. The AND function generates a new image that contains pixels that overlap between the red punctum and a green punctum. This new image can then be used to quantify colocalization with a third channel (blue). The coordinate and area information for colocalized puncta is then collected. Thus, this method is more accurate and versatile and runs more quickly than the circular approximation method.

(6) Experiment directory: the final user input field for the SynBot selection menu is to pick a folder that contains experimental images (experimental directory). The directory should include at least one subfolder. The subfolder(s) of the experimental directory should contain only the image files to be used by SynBot. We often divide experimental groups between the subfolders of the experimental directory, but this is not required and does not affect the analysis. It is also important that image names contain only one "." character preceding their file extension. The program uses this "." to separate the file extension from the image name and will not run properly if there are multiple.

SynBot outputs

A key feature of SynBot is that the users can easily check if the method is counting the correct objects as colocalized puncta because after each image is analyzed, SynBot creates a feedback image within the output folder with "_colocs" appended to the end of the image name. This "_colocs" image shows the input image with white circular overlays at the center position

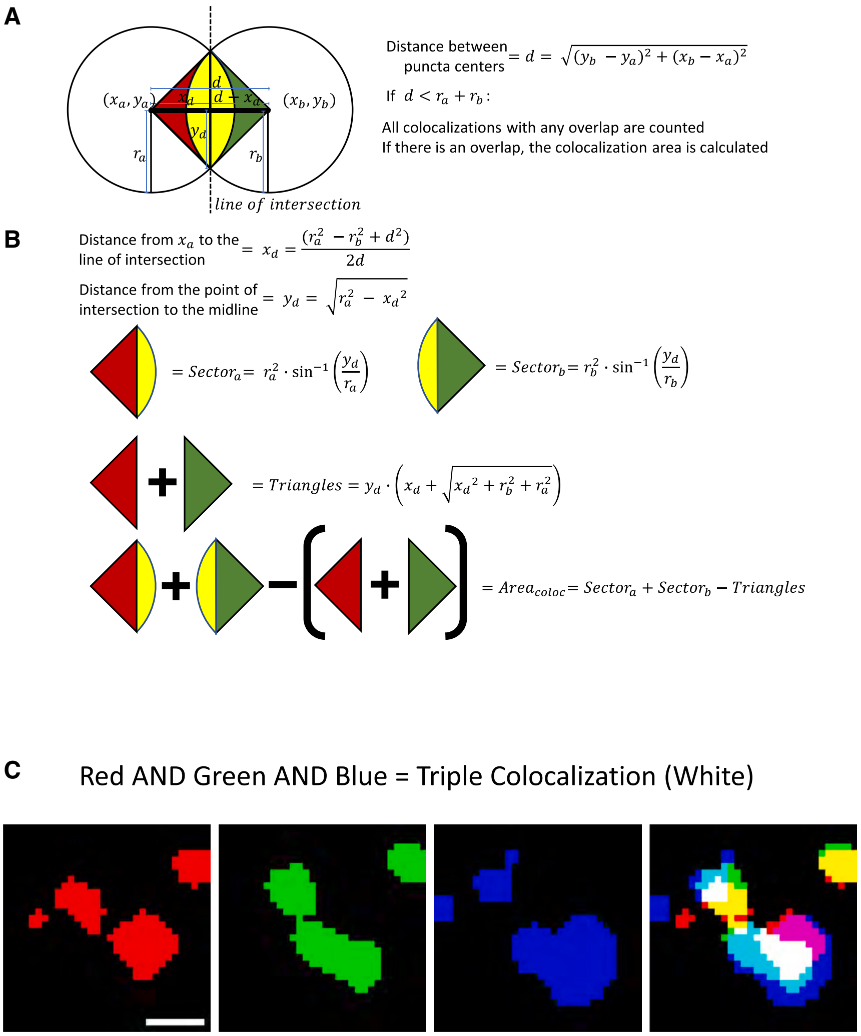

Figure 3. Detection and calculation of puncta colocalization

(A) SynBot detects colocalizations between synaptic puncta by first calculating the distance between the centers of the puncta and then determining if that distance is less than the sum of the radii of the puncta. If this distance is less than the sum of the radii, then the colocalization is recorded, and the area of colocalization is calculated.

(B) SynBot runs the following calculations for the area of colocalization area. (1) Calculates the distance between the center of the first puncta and the line of intersection. (2) Calculates the distances from the midpoint of the distance between the two puncta centers (d) to the intersection of the edges of the two puncta. (3) Calculates the area of the sector of the first puncta between the two intersection points. (4) Calculates the area of the sector for the second puncta. (5) Calculates the area of the rectangle formed by the two centers and intersection points, the sum of the two triangular portions of the sectors that are not part of the overlap. (6) The general form for the area of colocalization is the sum of the two sectors minus the two triangular portions outside of the overlap.

(C) Pictorial representation of the pixel overlap analysis mode where FIJI's image calculator is used to identify pixels that are part of both the red and green channels for a 2-channel colocalization or part of the red, green, and blue channels for a 3-channel colocalization. Scale bar represents 1 μm.

of each recorded colocalization. New SynBot users are encouraged to check these images after running the macro and adjust their analysis parameters if they see too few or too many calculated colocalizations that do not match what is evident by eye.

The primary output of SynBot, which contains the numbers of colocalized puncta per image, is the "summary" CSV file. This file contains the most commonly used measurements for synapse counting, such as the total numbers of red and green (and blue if triple colocalization is used) and the numbers of colocalized puncta from each image. The file also includes the thresholds and the settings for the minimal pixel size. To allow for the analysis of the cumulative properties of the synaptic puncta, the macro also saves the x and y coordinates and area of each individual puncta for the RGB and colocalized puncta into separate CSV files. These results are then ready for statistical analyses.

Quantification of synaptic colocalization by SynBot in simulated images

To validate SynBot's performance and compare the automated thresholding methods that are integrated within SynBot, we tested SynBot on simulated synapse images. We produced 20 simulated images with 667 red puncta, 666 green puncta, and 333 points where these puncta overlap to form a synapse. We then added a Gaussian noise background to each image with a mean and standard deviation proportional to the intensity histogram of the original image multiplied by 0.00, 0.25, 0.50, 0.75, or 1.00 to simulate images with varying noise levels (Figure 4A). This produces a set of images with no background (0.00), a background similar to what would be acquired in an experiment (0.25–0.75), and an excessive level of background (1.00). We then ran SynBot on these images using manual, ilastik, or SynQuant thresholding. Since the position of these simulated synapses was known, we computed the recall (true positive/(true positive + false negative)) (Figure 4B) and precision (true positive/(true positive + false positive)) (Figure 4C) for each method on our simulated images. These metrics are well suited to analyses where true positive, false positive, and false negative counts are obtained but true negative counts are not well defined (every point on the image without a synapse could be considered a true negative). We found that the recall and precision values were similar for each method. However, the methods all had decreased accuracy when the images contained excessive background. We found no significant differences between the recall or precision obtained with these thresholding methods when using a two-way ANOVA at each noise level (Figure S2).

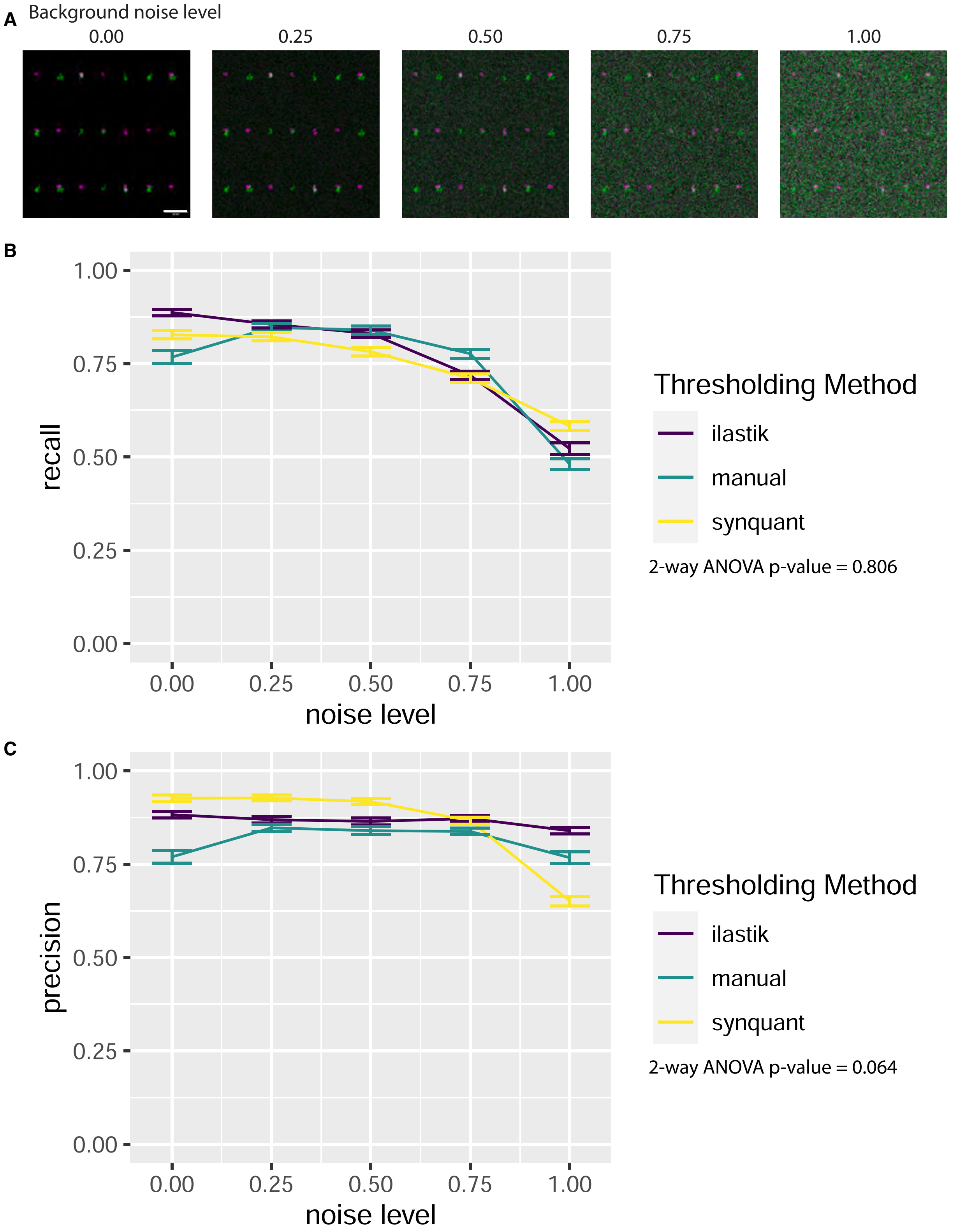

(legend on next page)

CellPress
OPEN ACCESS

Together, these analyses indicate that SynBot accurately counts synapses in realistic images containing low and high background noise.

Quantification of astrocyte-induced synaptogenesis by SynBot *in vitro*

To test SynBot's utility and accuracy, we next used a glia-free cortical neuronal culture system.[59,60] These neurons form few synapses when cultured alone; however, astrocytes secrete proteins that strongly promote excitatory and inhibitory synapse formation in these cultures.[59,60] The synaptogenic effects of astrocyte-conditioned medium (ACM) treatment are robust and promote a highly reproducible increase in the colocalization of pre- and post-synaptic puncta. This histological effect has been validated by numerous studies using EM and electrophysiology.[59,61–64] Therefore, to test the utility of SynBot and compare it to the Puncta Analyzer, here we used ACM treatment of purified cortical neuron cultures to induce synapse formation.

For these studies, neurons and astrocytes were individually prepared from post-natal day 1 (P1) Sprague-Dawley rat pups. First, we dissected the cerebral cortex and performed enzymatic digestion followed up by mechanical dissociation to obtain a single-cell suspension (Figure 5A). This single-cell suspension was then used to isolate neurons or astrocytes.

As illustrated in Figure 5B, neurons were purified from the single-cell suspensions first through a series of negative-panning steps to remove unwanted cell types in the following manner: first, using *Bandeiraea simplicifolia* lectin 1, we removed macrophages, and second, using an anti-mouse secondary antibody-coated plate and an anti-rat secondary antibody-coated plate, we removed potential non-specific Fc-binding cells, such as microglia and cell debris. After the negative selection, we then incubated the cell suspension on a positive-panning Petri dish coated with anti-L1CAM antibody. Neonatal cortical neurons highly express L1CAM, which is absent from other cortical cell types. Both excitatory and inhibitory neurons of the cortex are retained on the plate after incubation, and gentle washes are used to remove unbound cells. Finally, the neurons were collected from the positive-panning plate and plated on glass coverslips, which were coated with poly-D-lysine (PDL) and laminin.

To isolate astrocytes, we plated the cortical single-cell suspension onto a PDL-treated tissue culture flask. After 3 days *in vitro* (DIV3), we purified the astrocytes, which are tightly attached to the flask, by vigorously shaking so that less-adherent cells are removed. To collect the ACM, we incubated the astrocytes with minimal media for 4 days to promote protein secretion. We collected the ACM and concentrated it using centrifugal concentrator tubes (Figure 5C). After protein quantification, we applied 50 or 100 μg/mL ACM to the glia-free neuronal cultures at DIV8 and DIV11 to induce excitatory and inhibitory synapse formation, respectively (Figure 5D).

To label excitatory and inhibitory synapses in culture, we fixed the DIV13 neuronal cultures and immunostained the synapses with pre- and post-synaptic markers. To identify excitatory synapses, we used the pre-synaptic active zone marker Bassoon together with the excitatory post-synaptic marker Homer1 (Figure 5E). To visualize inhibitory synapses, we used Bassoon in combination with the inhibitory post-synapse marker gephyrin (Figure 5G). For image acquisition, we took single-focal-plane images using an inverted Olympus FV3000 confocal laser scanning microscope with a 60× oil immersion objective. The images were taken in 1,024 × 1,024 resolution with a 2× digital zoom. To identify individual neurons, we used the DAPI channel, and we imaged only the neuronal cell bodies at least two cell diameters distant from other neurons. We used this strategy to avoid overlapping cells and to use an unbiased method to select the neurons to image. We imaged at least 20 cells for each condition, and the laser power for each channel was adjusted such that the signal for each channel was bright without many saturated pixels.

To determine the efficiency of SynBot to detect pre- and post-synaptic colocalization, we analyzed images from cortical neurons treated with ACM or cultured in growth media only (named here as control) using the manual, ilastik, and SynQuant thresholding methods of SynBot. We compared these results to analyses done by its predecessor, Puncta Analyzer (Figures 5E–5H and S3). To aid these comparisons, we normalized the number of excitatory synapses between the ACM-treated neurons to the control values. As expected, ACM induced a significant increase in synapse numbers (∼1.5-fold) with each of the analysis methods used (Student's t test $p = 0.005$, $p = 0.004$, $p = 0.010$, and $p = 0.0150$ for the Puncta Analyzer, SynBot manual, SynBot ilastik, and SynBot SynQuant, respectively) (Figure 5F). Similarly, we found a 2-fold increase in the number of inhibitory synapses when neurons were treated with ACM compared to the control (Student's t test $p < 0.001$ for each analysis method) (Figure 5H). These results indicate that SynBot is equally efficient in detecting both types of synaptic contacts as the Puncta Analyzer. Moreover, these results show that the ilastik and SynQuant automated thresholding methods can be used to determine changes in synapse numbers.

To determine whether manual or automated thresholding affected the numbers of puncta identified, we also compared the raw numbers of green (pre-synaptic marker), red (post-synaptic marker), and colocalizations identified between the Puncta Analyzer, SynBot manual, SynBot ilastik, and SynBot SynQuant. When compared to the other methods, SynBot ilastik consistently identified more green, red, and colocalized puncta

Figure 4. SynBot quantifies simulated synapse data with high recall and precision

(A) Simulated synapse images with differing levels of Gaussian noise background. Synapses were copied from VGluT1-PSD95 a2d1 WT and KO images and then pasted onto Gaussian noise background with a mean and standard deviation proportional to the same values in the original image multiplied by the given noise level (0.00, 0.25, 0.50, 0.75, 1.00). Scale bar represents 20 μm.

(B) Recall (true positive/(true positive + false negative)) obtained using SynBot with manual, ilastik, and SynQuant thresholding methods. 20 images were analyzed at each noise level. Error bars represent 1 standard error of the mean.

(C) Precision (true positive/(true positive + false positive)) obtained using SynBot with manual, ilastik, and SynQuant thresholding methods. 20 images were analyzed at each noise level. Error bars represent 1 standard error of the mean.

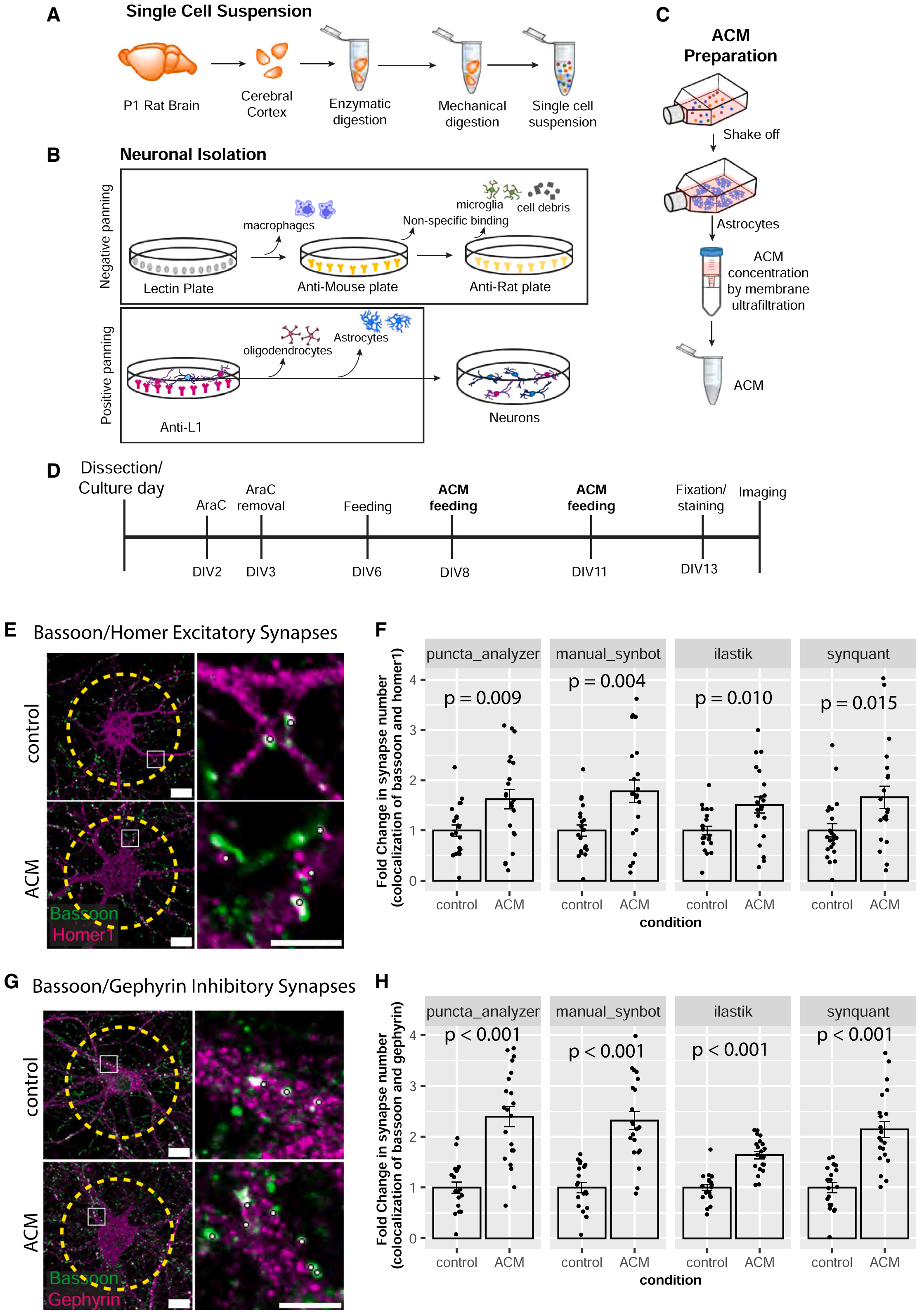

(legend on next page)

from the images, regardless of the antibodies used or the treatment conditions (Figure S2). This result is consistent with ilastik's tendency to detect more individual objects (here puncta) based on 37 different image features. In contrast, manual thresholding only uses intensity cutoffs and minimal puncta size as features for detection. Two-way ANOVA tests found significant differences in the raw numbers of colocalized synapses detected by each of the analysis methods for the *in vitro* excitatory and inhibitory datasets shown in Figure 5 (see Figure S2). When the number of colocalized puncta detected was normalized to the appropriate control conditions, however, there were no significant differences between the 4 thresholding methods. Together, these results show that SynBot, with its user-friendly and time-saving features, efficiently detects excitatory and inhibitory synaptic contacts *in vitro*.

Quantification of *in vivo* excitatory synapse numbers using SynBot

After validating the use of SynBot *in vitro*, we next tested its utility in quantifying synapse numbers from mouse brain tissue sections. Immunohistochemistry of brain tissue sections is a common method for synapse number quantification.[40,41,43,45,50,60] However, these analyses have the considerable added complexity of a 3D tissue section that should be imaged. Moreover, thick tissue sections from the brain inherently have more background signals, which can impair the accuracy of the analyses. Finally, antibody penetration into the tissue sections is an important consideration while optimizing synapse staining and imaging procedures. Before using SynBot or any other method, the experimenters should optimize their staining and imaging procedures. The optimized synapse staining and imaging procedure we have used can be found in the STAR Methods and protocols.io (https://www.protocols.io/view/synbot-protocols-3byl4qewjvo5/v2).

To test SynBot's efficacy and accuracy in quantifying synapse numbers *in vivo*, we next analyzed images that were previously acquired and reported in a study by Risher and colleagues in 2018.[50] This paper showed that the neuronal thrombospondin/gabapentin receptor $\alpha2\delta$-1 is crucial for proper excitatory synapse formation in the developing mouse cortex.[50,62,65] Risher et al. showed that in $\alpha2\delta$-1 knockout (KO) animals, there is a significant decrease in VGluT1/PSD95 synapse numbers compared to wild-type (WT) controls.[50] Risher et al. validated these histological findings of impaired synaptogenesis using electrophysiology and EM, which also showed a strong decrease in excitatory synapse function and number, respectively.

To perform these experiments, mice were transcardially perfused with 4% paraformaldehyde, and brains were collected, frozen, and cryosectioned (Figure 6A). Intracortical excitatory synapses were marked with the pre- and post-synaptic markers specific for these connections, namely VGluT1 and PSD95 (Figure 6B). Images were acquired using a Leica SP5 confocal microscope. A z stack of 15 optical planes was acquired using a 0.34 μm z-step to image ~5 μm of the sample. Images were acquired such that confocal settings were optimized on control conditions to minimize oversaturated pixels. For detailed procedures, see the STAR Methods and protocols.io (https://www.protocols.io/view/synbot-protocols-3byl4qewjvo5/v2).

We used this well-validated dataset to test SynBot's efficacy for quantifying synapse numbers in mouse brain tissue images. We reanalyzed the P21 $\alpha2\delta$-1 WT and KO images previously published in Risher et al. using the SynBot manual, ilastik, and SynQuant thresholding methods and compared these to the results Risher et al. obtained using the Puncta Analyzer (Figure 6C). All 4 methods found a significant ~50% decrease in the number of synapses in $\alpha2\delta$-1 KO animals when compared to WT littermates (linear mixed-effects model, $p < 0.001$ for each method). In our hands, a pre-trained ilastik model implemented in SynBot or SynQuant automated thresholding was able to perform as well as a highly trained manual user (Figures 6C and S2). As we saw with the *in vitro* datasets, there were significant differences between the raw numbers of colocalized puncta detected by the 4 thresholding methods but not after normalization to the WT control data (two-way ANOVA with a p value cutoff of 0.05; Figure S2C). Quantification using SynBot manual, ilastik, or SynQuant thresholding recapitulated the previously validated finding that $\alpha2\delta$-1 KO mice have fewer intracortical synapses than WT littermates, which was also previously confirmed by EM and electrophysiology.[50] This demonstrates that SynBot can be successfully applied to brain tissue images and that the thresholding methods packaged in SynBot can be used to compare different strategies quickly.

Altogether, SynBot is a fast, reliable, user-friendly, and versatile software to study synapse density *in vitro* and *in vivo*, overcoming previous constraints of the Puncta Analyzer.

Figure 5. SynBot quantifies astrocyte-induced synapse formation *in vitro*
(A) Scheme of single-cell suspension generation from P1 rat pup cortices.
(B) Scheme of neuronal isolation by immunopanning first through a series of negative-panning steps and then through positive panning with anti-L1CAM antibody.
(C) Scheme of astrocyte purification and astrocyte-conditioned medium (ACM) preparation.
(D) Timeline of neuronal feeding schedule and ACM treatment.
(E) Representative images of excitatory synaptic puncta stained with pre-synaptic marker Bassoon and post-synaptic marker Homer1. Scale bars represent 15 μm for full image and 5 μm for zoom in. Yellow dashed line represents the ROI analyzed. White dots represent counted synapses.
(F) Example of quantification of excitatory synaptic colocalization between control and ACM using the Puncta Analyzer, SynBot manual thresholding, SynBot ilastik, or SynBot SynQuant thresholding. Student's t test, 20 neurons per treatment. Error bars represent 1 standard error of the mean.
(G) Representative images of inhibitory synaptic puncta stained with pre-synaptic marker Bassoon and post-synaptic marker Gephyrin. Scale bars represent 15 μm for full image and 5 μm for zoom in. Yellow dashed line represents the ROI analyzed. White dots represent counted synapses.
(H) Example of quantification of inhibitory synaptic colocalization between control and ACM using the Puncta Analyzer, SynBot manual thresholding, SynBot ilastik thresholding, or SynBot SynQuant thresholding. Student's t test, 20 neurons per treatment. Error bars represent 1 standard error of the mean.
See also Figures S2 and S3 and Table S2.

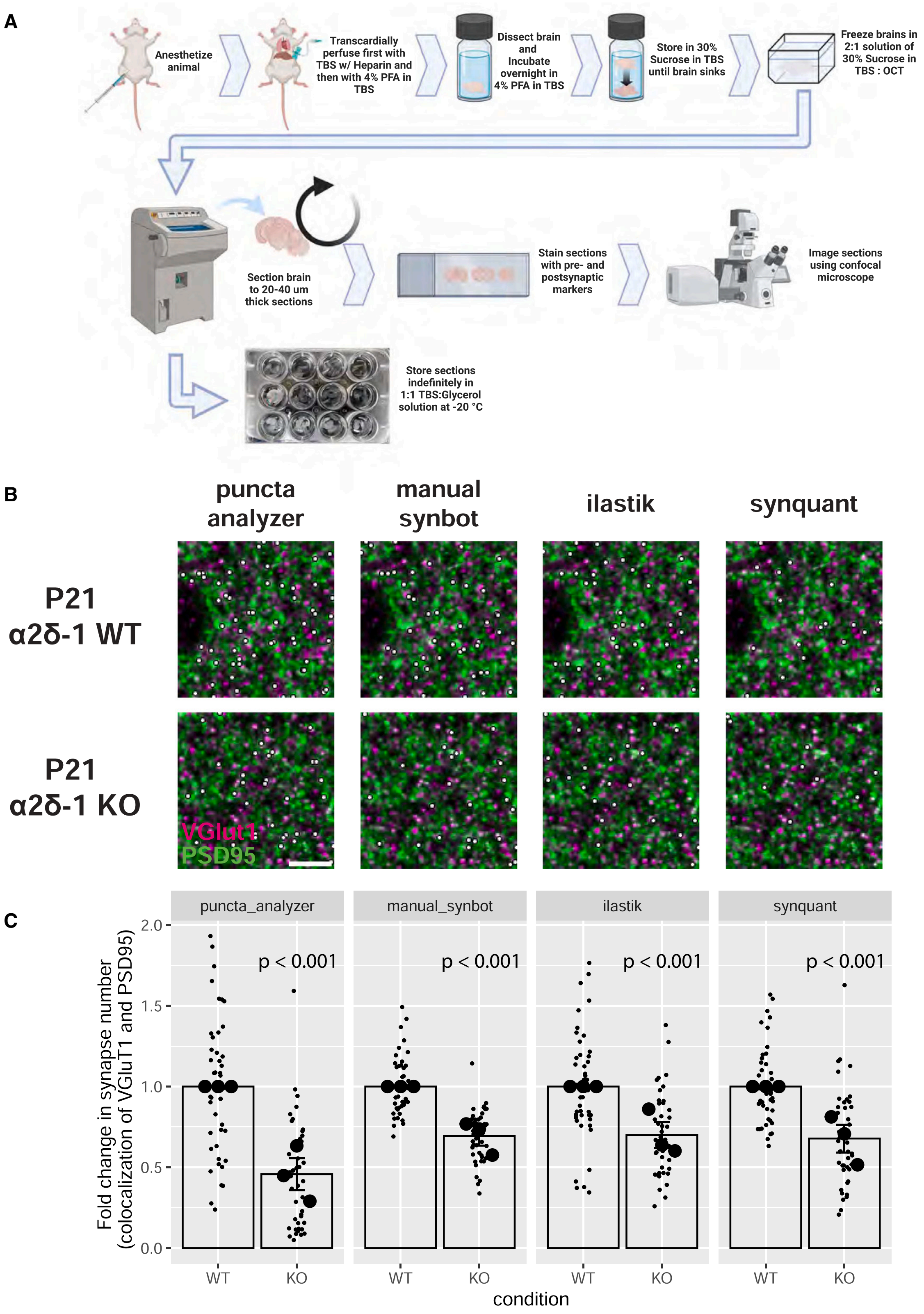

(legend on next page)

DISCUSSION

Quantification of synapse numbers using immunohistochemistry is a favored technique for its ability to label synapses quickly and easily. However, this rapid experimental protocol is often paired with slow analysis methods that are difficult to learn and result in variability between users. SynBot overcomes many of the shortcomings of its predecessor by being easy to learn, with built-in and online instructions, and easy to use, with a user interface that simplifies and streamlines user input. SynBot also allows unsupervised analyses through its automated methods. Importantly, SynBot collects all the data extracted from synaptic images by recording the position and area of each individual punctum from each image channel. These data can be used for other forms of analyses, such as the count and sizes of individual synaptic puncta or the spatial relationship in their distribution across the tissue imaged.

One of the most challenging aspects of analyzing these data is the subjective nature of the manual thresholding used in the Puncta Analyzer. The 9 thresholding modes available in SynBot address this by providing the user with simple approaches like a fixed threshold value, streamlined manual thresholding, and the complex machine-learning-based algorithm of ilastik and the probability-based algorithm of SynQuant. We have found ilastik to be particularly suited to this workflow, as a short training session in ilastik is sufficient to analyze images accurately and reproducibly without the need for further user input.

The advantage of implementing SynBot in the ImageJ macro language is the ease with which it can be changed by users to apply to a wider variety of experimental questions. All the source code for the macro is available on our lab GitHub site (https://github.com/Eroglu-Lab/Syn_Bot) and can be edited within FIJI itself without the need for software development programs or in-depth computer science experience. There were only a few parts of SynBot that could not be implemented in the ImageJ macro language (circular approximation colocalization, some dialogue menus, ilastik and SynQuant integration) and required Java programming. These sections are stored in separate GitHub repositories, ilastik4ij_Syn_Bot (https://github.com/Eroglu-Lab/Syn_Bot/blob/main/ilastik4ij_Syn_Bot-1.8.2-SNAPSHOT.jar) and SynQuantSimple (https://github.com/freemanwyz/SynQuantSimple), and are freely available for download and can be edited by users with experience in Java programming.

Another benefit of SynBot over previous analysis programs is its speed. When analyzing the 100 simulated images shown in Figure 4, for example, SynBot analysis took 24 min for manual thresholding, 10 min for training and 50 min for ilastik thresholding, and 4 min for SynQuant thresholding. Unlike manual thresholding, the time spent while ilastik or SynQuant runs requires no user input. The speed of SynQuant makes it well suited for running multiple times using different parameters, making it easy to troubleshoot and optimize accuracy for a given dataset.

We found in our analysis that each thresholding method showed a similar effect size when experimental conditions were normalized to controls. However, the raw synapse counts varied by analysis method (Figure S2). This difference was particularly pronounced for *in vivo* data due to the greater complexity of this sample type. Another source of discrepancy between thresholding methods is the exclusion of certain synapses when a given thresholding method results in either channel being too small at that location.

With all of these features, researchers will be able to rapidly screen experimental conditions that alter synapse numbers and can move on to in-depth structural and mechanistic investigations with other methods, such as electrophysiology, super-resolution microscopy, or EM. Therefore, SynBot will aid in answering many outstanding questions related to synapse development and maintenance.

Limitations of the study

There are a few key conditions necessary for SynBot to be successfully applied. Firstly, the signals being analyzed need to be punctate rather than diffuse since SynBot performs object-based colocalizations and must be able to identify discrete objects. Similarly, objects within the images must be non-overlapping to be independently counted. This object-based colocalization is most appropriate when the position of synaptic compartments is changing to alter the number of structural synapses (seen as colocalizations). If the abundance of a synaptic marker or its association with synapses is changing, intensity measurements or pixel-wise colocalization on unthresholded raw images may be more appropriate (see https://imagej.net/imaging/colocalization-analysis for further discussion of different colocalization methods).

SynBot relies on synaptic puncta position rather than raw intensity. Thus, we can use thresholding methods such as ilastik and SynQuant, which apply different values to each image to separate the foreground from the background. However, if needed, SynBot can also apply the "fixed value" thresholding, which allows users to apply the same exact threshold value to all images. An important limitation of this fixed thresholding method is that it would only be useful for datasets with images containing minimal background noise, such as *in vitro* synapse staining.

Figure 6. SynBot quantifies reduced α2δ-1 KO synapse numbers *in vivo*

(A) Workflow for harvesting mouse brain tissue and preparing it for synapse imaging.

(B) Representative images of synapses from layer 2/3 of primary visual cortex in WT versus α2δ-1 KO mice at P21. Magenta: VGluT1; green: PSD95. Synapses identified by each thresholding method are marked by small white dots. Scale bar represents 5 μm.

(C) Quantification of VGluT1-PSD95 colocalization using the Puncta Analyzer, SynBot manual thresholding, SynBot ilastik thresholding, or SynBot SynQuant thresholding. Colocalized puncta counts were normalized to the WT average for each experimental pair. A linear mixed-effects model was used to account for multiple images per animal. Mouse averages are shown as large black dots ($n = 3$ mice per condition), with individual images shown as small gray dots (3 fields of view per animal with 5 z projections per field of view). Error bars represent 1 standard error of the mean.

See also Figures S1 and S2 and Table S2.

While theoretically possible, in practice, staining intensities and background may vary even within a single experiment enough to impair the use of fixed values. We strongly recommend using methods like ilastik or SynQuant instead because they have the flexibility to adjust to the varying image brightness. In particular, SynQuant thresholds images using a statistical method that provides consistency between different images. Thus, these automated methods make these analyses much more accessible for untrained new users than manual thresholding.

SynBot relies on RGB image conversion to keep track of the different image color channels. This limits SynBot analysis to a maximum of 3 colors and converts each image channel to an 8-bit format. This reduction in bit depth from the 16-bit images acquired by many modern microscopes reduces the possible thresholding values from 65,536 in a 16-bit image to 256 in an 8-bit image. For the synapse analysis experiments shown here, 256 possible values are adequate for thresholding, but certain analyses may benefit from dividing the image intensity range into a greater number of bins. It should be noted that SynBot has no limitations for image resolution (the number of pixels representing a given area of space) and that this bit depth conversion has no impact on resolution.

SynBot only works on single optical sections or z stack projections and is unable to fully incorporate 3D synapse structures, as is done in other software, such as Imaris. Despite these caveats, SynBot is widely applicable to any object-based colocalization analysis for synapses or any other set of imaging markers. SynBot can tailor its analysis to many experimental questions. However, for neurite tracing synapse analysis or 3D constructions, alternative methods should be used.[51,52] Similarly, if the user needs to analyze multiple regions from an image, then they should perform repetitive analysis to follow that experimental design.

It is also important to note that the accuracy of synapse number analyses is heavily dependent on the quality of staining and imaging. Moreover, proper markers should be used to label synapses. Some common mistakes are to use an axonal or dendritic marker, such as GAD65 or GAD67 (for inhibitory axons) or MAP2 (for neuronal dendrites), as one of the synaptic compartments. These kinds of markers are often extrasynaptically localized and widely distributed, thus yielding colocalization with other markers even if they were not at an actual synapse.

Similarly, SynBot requires careful troubleshooting, even when using fully automated thresholding methods. Users should always visually inspect the counted synapses using the "colocs" output images and verify the thresholding performance by viewing the "red_thresholded" and "green_thresholded" output images. It is also advisable to include control conditions whenever possible, such as perturbations known to change synapse numbers. As with any image analysis method, improper use can lead to misleading results.

RESOURCE AVAILABILITY

Lead contact

Further information and requests for resources and reagents should be directed to and will be fulfilled by the lead contact, Cagla Eroglu (cagla.eroglu@duke.edu).

Materials availability

This study did not generate new unique reagents.

Data and code availability

- Microscopy data and associated synapse counts reported in this paper are available on Zenodo at the following DOI: https://doi.org/10.5281/zenodo.12191805.
- All original code has been deposited at our lab GitHub site (https://github.com/Eroglu-Lab/Syn_Bot) and is publicly available on Zenodo at the following DOI: https://doi.org/10.5281/zenodo.12571789.
- Any additional information required to reanalyze the data reported in this paper is available from the lead contact upon request.

ACKNOWLEDGMENTS

We thank Christabel Tan, M. Pia Rodriguez-Salazar, Dr. Oluwadamilola Lawal, Nicholas Brose, Gabrielle Sejourne, Kavya Raghunathan, and other Eroglu lab members for critical feedback about the manuscript. This work was supported by NIH R01 funding (AG059409 and NS102237), BRAIN Initiative funding (U19NS123719), Chan Zuckerberg Initiative Neurodegeneration Challenge Network Collaborative grants (2018-191999 and DAF2021-237435), the Holland-Trice Scholars Award for high-risk/high-impact discovery in research in Basic Brain Science and Brain Diseases (C.E.), and the joint efforts of MJFF and the Aligning Science Across Parkinson's (ASAP) initiative. MJFF administers the grant (ASAP-020607 to C.E.) on behalf of ASAP and the Michael J. Fox Foundation. J.T.S. was supported by NIH funding (F31NS134252). D.I. was supported by the Pew Latin American Fellows Program in the Biomedical Sciences and the Foerster-Bernstein Post-Doctoral Fellowship Program for Women in STEM. J.J.R. was supported by NIH funding (NS102237-S1 and F31NS125985). Y.W. was supported by NIH funding (R01MH110504). W.C.R. was supported by NIH funding (R15MH126345). C.E. is an HHMI Investigator. The graphical abstract, cartoon of a synapse in Figure 1, and diagram of mouse brain tissue staining in Figure 5 were created using BioRender.com.

AUTHOR CONTRIBUTIONS

Conceptualization, J.T.S. and C.E.; software, J.T.S., J.J.R., and Y.W.; investigation, J.J.R., W.C.R., and D.I.; writing – original draft, J.T.S., J.J.R., D.I., and C.E.; writing – review & editing, J.T.S., J.J.R., D.I., and C.E.

DECLARATION OF INTERESTS

The authors declare no competing interests.

STAR★METHODS

Detailed methods are provided in the online version of this paper and include the following:

- KEY RESOURCES TABLE
- EXPERIMENTAL MODEL AND STUDY PARTICIPANT DETAILS
  - Animals
- METHOD DETAILS
  - *In vitro* synapse assay with astrocyte conditioned media
  - Mouse brain tissue synapse assay
  - Image analysis
- QUANTIFICATION AND STATISTICAL ANALYSIS

SUPPLEMENTAL INFORMATION

Supplemental information can be found online at https://doi.org/10.1016/j.crmeth.2024.100861.

Received: July 12, 2023
Revised: April 25, 2024
Accepted: August 16, 2024
Published: September 9, 2024

REFERENCES

1. Farhy-Tselnicker, I., and Allen, N.J. (2018). Astrocytes, neurons, synapses: a tripartite view on cortical circuit development. Neural Dev. *13*, 7. https://doi.org/10.1186/S13064-018-0104-Y.
2. Cadwell, C.R., Bhaduri, A., Mostajo-Radji, M.A., Keefe, M.G., and Nowakowski, T.J. (2019). Development and Arealization of the Cerebral Cortex. Neuron *103*, 980–1004. https://doi.org/10.1016/J.NEURON.2019.07.009.
3. Pan, Y., and Monje, M. (2020). Activity Shapes Neural Circuit Form and Function: A Historical Perspective. J. Neurosci. *40*, 944–954. https://doi.org/10.1523/JNEUROSCI.0740-19.2019.
4. Luo, L. (2021). Architectures of Neuronal Circuits. Science *373*, eabg7285. https://doi.org/10.1126/SCIENCE.ABG7285.
5. Batool, S., Raza, H., Zaidi, J., Riaz, S., Hasan, S., and Syed, N.I. (2019). Synapse formation: From cellular and molecular mechanisms to neurodevelopmental and neurodegenerative disorders. J. Neurophysiol. *121*, 1381–1397. https://doi.org/10.1152/jn.00833.2018.
6. Rizalar, F.S., Roosen, D.A., and Haucke, V. (2021). A Presynaptic Perspective on Transport and Assembly Mechanisms for Synapse Formation. Neuron *109*, 27–41. https://doi.org/10.1016/J.NEURON.2020.09.038.
7. Groc, L., and Choquet, D. (2020). Linking glutamate receptor movements and synapse function. Science *368*, eaay4631. https://doi.org/10.1126/SCIENCE.AAY4631.
8. Newschaffer, C.J., Croen, L.A., Daniels, J., Giarelli, E., Grether, J.K., Levy, S.E., Mandell, D.S., Miller, L.A., Pinto-Martin, J., Reaven, J., et al. (2020). An Overview of the Main Genetic, Epigenetic and Environmental Factors Involved in Autism Spectrum Disorder Focusing on Synaptic Activity. Int. J. Mol. Sci. *21*, 8290. https://doi.org/10.3390/IJMS21218290.
9. Pocklington, A.J., O'Donovan, M., and Owen, M.J. (2014). The synapse in schizophrenia. Eur. J. Neurosci. *39*, 1059–1067. https://doi.org/10.1111/EJN.12489.
10. Lane-Donovan, C., and Herz, J. (2017). ApoE, ApoE Receptors, and the Synapse in Alzheimer's Disease. Trends Endocrinol. Metabol. *28*, 273–284. https://doi.org/10.1016/J.TEM.2016.12.001.
11. Rizo, J., and Xu, J. (2015). The Synaptic Vesicle Release Machinery. Annu. Rev. Biophys. *44*, 339–367. https://doi.org/10.1146/ANNUREV-BIOPHYS-060414-034057.
12. Rusakov, D.A., Savtchenko, L.P., Zheng, K., and Henley, J.M. (2011). Shaping the synaptic signal: molecular mobility inside and outside the cleft. Trends Neurosci. *34*, 359–369. https://doi.org/10.1016/J.TINS.2011.03.002.
13. Luján, R., Shigemoto, R., and López-Bendito, G. (2005). Glutamate and GABA receptor signalling in the developing brain. Neuroscience *130*, 567–580. https://doi.org/10.1016/J.NEUROSCIENCE.2004.09.042.
14. Vyklicky, V., Korinek, M., Smejkalova, T., Balik, A., Krausova, B., Kaniakova, M., Lichnerova, K., Cerny, J., Krusek, J., Dittert, I., et al. (2014). Structure, function, and pharmacology of NMDA receptor channels. Physiol. Res. *63*, S191–S203. https://doi.org/10.33549/PHYSIOLRES.932678.
15. Diering, G.H., and Huganir, R.L. (2018). The AMPA Receptor Code of Synaptic Plasticity. Neuron *100*, 314–329. https://doi.org/10.1016/J.NEURON.2018.10.018.
16. Chen, H., Tang, A.H., and Blanpied, T.A. (2018). Subsynaptic spatial organization as a regulator of synaptic strength and plasticity. Curr. Opin. Neurobiol. *51*, 147–153. https://doi.org/10.1016/J.CONB.2018.05.004.
17. Iasevoli, F., Tomasetti, C., and de Bartolomeis, A. (2013). Scaffolding proteins of the post-synaptic density contribute to synaptic plasticity by regulating receptor localization and distribution: relevance for neuropsychiatric diseases. Neurochem. Res. *38*, 1–22. https://doi.org/10.1007/S11064-012-0886-Y.
18. Akin, O., and Zipursky, S.L. (2020). Activity regulates brain development in the fly. Curr. Opin. Genet. Dev. *65*, 8–13. https://doi.org/10.1016/J.GDE.2020.04.005.
19. Andreae, L.C., and Burrone, J. (2014). The role of neuronal activity and transmitter release on synapse formation. Curr. Opin. Neurobiol. *27*, 47–52. https://doi.org/10.1016/J.CONB.2014.02.008.
20. Chen, L.F., Zhou, A.S., and West, A.E. (2017). Transcribing the connectome: roles for transcription factors and chromatin regulators in activity-dependent synapse development. J. Neurophysiol. *118*, 755–770. https://doi.org/10.1152/JN.00067.2017.
21. Shen, K., and Scheiffele, P. (2010). Genetics and cell biology of building specific synaptic connectivity. Annu. Rev. Neurosci. *33*, 473–507. https://doi.org/10.1146/ANNUREV.NEURO.051508.135302.
22. Sanes, J.R., and Zipursky, S.L. (2020). Synaptic Specificity, Recognition Molecules, and Assembly of Neural Circuits. Cell *181*, 536–556. https://doi.org/10.1016/J.CELL.2020.04.008.
23. Jain, S., and Zipursky, S.L. (2023). Temporal control of neuronal wiring. Semin. Cell Dev. Biol. *142*, 81–90. https://doi.org/10.1016/J.SEMCDB.2022.05.012.
24. Farhy-Tselnicker, I., Boisvert, M.M., Liu, H., Dowling, C., Erikson, G.A., Blanco-Suarez, E., Farhy, C., Shokhirev, M.N., Ecker, J.R., and Allen, N.J. (2021). Activity-dependent modulation of synapse-regulating genes in astrocytes. Elife *10*, e70514. https://doi.org/10.7554/ELIFE.70514.
25. Wilton, D.K., Dissing-Olesen, L., and Stevens, B. (2019). Neuron-Glia Signaling in Synapse Elimination. Annu. Rev. Neurosci. *42*, 107–127. https://doi.org/10.1146/ANNUREV-NEURO-070918-050306.
26. Chung, W.S., Allen, N.J., and Eroglu, C. (2015). Astrocytes Control Synapse Formation, Function, and Elimination. Cold Spring Harbor Perspect. Biol. *7*, a020370. https://doi.org/10.1101/CSHPERSPECT.A020370.
27. Bonetto, G., Belin, D., and Káradóttir, R.T. (2021). Myelin: A gatekeeper of activity-dependent circuit plasticity? Science *374*, eaba6905. https://doi.org/10.1126/SCIENCE.ABA6905.
28. Sancho, L., Contreras, M., and Allen, N.J. (2021). Glia as sculptors of synaptic plasticity. Neurosci. Res. *167*, 17–29. https://doi.org/10.1016/J.NEURES.2020.11.005.
29. Allen, N.J., and Eroglu, C. (2017). Cell Biology of Astrocyte-Synapse Interactions. Neuron *96*, 697–708. https://doi.org/10.1016/J.NEURON.2017.09.056.
30. Baldwin, K.T., and Eroglu, C. (2017). Molecular mechanisms of astrocyte-induced synaptogenesis. Curr. Opin. Neurobiol. *45*, 113–120. https://doi.org/10.1016/J.CONB.2017.05.006.
31. Tan, C.X., Burrus Lane, C.J., and Eroglu, C. (2021). Role of astrocytes in synapse formation and maturation. Curr. Top. Dev. Biol. *142*, 371–407. https://doi.org/10.1016/BS.CTDB.2020.12.010.
32. Watanabe, S., Trimbuch, T., Camacho-Pérez, M., Rost, B.R., Brokowski, B., Söhl-Kielczynski, B., Felies, A., Davis, M.W., Rosenmund, C., and Jorgensen, E.M. (2014). Clathrin regenerates synaptic vesicles from endosomes. Nature *515*, 228–233. https://doi.org/10.1038/nature13846.
33. Begemann, I., and Galic, M. (2016). Correlative Light Electron Microscopy: Connecting Synaptic Structure and Function. Front Synaptic Neurosci *8*, 28. https://doi.org/10.3389/fnsyn.2016.00028.
34. Staffler, B., Berning, M., Boergens, K.M., Gour, A., Smagt, P.v.d., and Helmstaedter, M. (2017). SynEM, automated synapse detection for connectomics. Elife *6*, e26414. https://doi.org/10.7554/ELIFE.26414.
35. Dorkenwald, S., Schubert, P.J., Killinger, M.F., Urban, G., Mikula, S., Svara, F., and Kornfeld, J. (2017). Automated synaptic connectivity inference for volume electron microscopy. Nat. Methods *14*, 435–442. https://doi.org/10.1038/nmeth.4206.
36. Chandrasekaran, S., Navlakha, S., Audette, N.J., McCreary, D.D., Suhan, J., Bar-Joseph, Z., and Barth, A.L. (2015). Unbiased, High-Throughput Electron Microscopy Analysis of Experience-Dependent Synaptic Changes in the Neocortex. J. Neurosci. *35*, 16450–16462. https://doi.org/10.1523/JNEUROSCI.1573-15.2015.
37. Segev, A., Garcia-Oscos, F., and Kourrich, S. (2016). Whole-cell Patch-clamp Recordings in Brain Slices. J. Vis. Exp. *2016*, 54024. https://doi.org/10.3791/54024.

38. Ippolito, D.M., and Eroglu, C. (2010). Quantifying Synapses: an Immunocytochemistry-based Assay to Quantify Synapse Number. JoVE *45*, e2270. https://doi.org/10.3791/2270.

39. Maidorn, M., Rizzoli, S.O., and Opazo, F. (2016). Tools and limitations to study the molecular composition of synapses by fluorescence microscopy. Biochem. J. *473*, 3385–3399. https://doi.org/10.1042/BCJ20160366.

40. Blanco-Suarez, E., Liu, T.F., Kopelevich, A., and Allen, N.J. (2018). Astrocyte-Secreted Chordin-like 1 Drives Synapse Maturation and Limits Plasticity by Increasing Synaptic GluA2 AMPA Receptors. Neuron *100*, 1116–1132.e13. https://doi.org/10.1016/J.NEURON.2018.09.043.

41. Stogsdill, J.A., Ramirez, J., Liu, D., Kim, Y.H., Baldwin, K.T., Enustun, E., Ejikeme, T., Ji, R.R., and Eroglu, C. (2017). Astrocytic neuroligins control astrocyte morphogenesis and synaptogenesis. Nature *551*, 192–197. https://doi.org/10.1038/nature24638.

42. Holt, L.M., Hernandez, R.D., Pacheco, N.L., Torres Ceja, B., Hossain, M., and Olsen, M.L. (2019). Astrocyte morphogenesis is dependent on BDNF signaling via astrocytic TrkB. Elife *8*, e44667. https://doi.org/10.7554/ELIFE.44667.

43. Nguyen, A.Q., Sutley, S., Koeppen, J., Mina, K., Woodruff, S., Hanna, S., Vengala, A., Hickmott, P.W., Obenaus, A., and Ethell, I.M. (2020). Astrocytic Ephrin-B1 Controls Excitatory-Inhibitory Balance in Developing Hippocampus. J. Neurosci. *40*, 6854–6871. https://doi.org/10.1523/JNEUROSCI.0413-20.2020.

44. Glebov, O.O., Cox, S., Humphreys, L., and Burrone, J. (2016). Neuronal activity controls transsynaptic geometry. Sci. Rep. *6*, 22703–22711. https://doi.org/10.1038/srep22703.

45. Baldwin, K.T., Tan, C.X., Strader, S.T., Jiang, C., Savage, J.T., Elorza-Vidal, X., Contreras, X., Rülicke, T., Hippenmeyer, S., Estévez, R., et al. (2021). HepaCAM controls astrocyte self-organization and coupling. Neuron *109*, 2427–2442.e10. https://doi.org/10.1016/j.neuron.2021.05.025.

46. Verstraelen, P., Garcia-Diaz Barriga, G., Verschuuren, M., Asselbergh, B., Nuydens, R., Larsen, P.H., Timmermans, J.P., and De Vos, W.H. (2020). Systematic Quantification of Synapses in Primary Neuronal Culture. iScience *23*, 101542. https://doi.org/10.1016/j.isci.2020.101542.

47. McNamara, G., Difilippantonio, M., Ried, T., and Bieber, F.R. (2017). Microscopy and Image Analysis. Curr. Protoc. Hum. Genet. *94*, 4.4.1–4.4.89. https://doi.org/10.1002/CPHG.42.

48. Dani, A., Huang, B., Bergan, J., Dulac, C., and Zhuang, X. (2010). Superresolution Imaging of Chemical Synapses in the Brain. Neuron *68*, 843–856. https://doi.org/10.1016/J.NEURON.2010.11.021.

49. Risher, W.C., Patel, S., Kim, I.H., Uezu, A., Bhagat, S., Wilton, D.K., Pilaz, L.J., Singh Alvarado, J., Calhan, O.Y., Silver, D.L., et al. (2014). Astrocytes refine cortical connectivity at dendritic spines. Elife *3*, e04047. https://doi.org/10.7554/ELIFE.04047.

50. Risher, W.C., Kim, N., Koh, S., Choi, J.E., Mitev, P., Spence, E.F., Pilaz, L.J., Wang, D., Feng, G., Silver, D.L., et al. (2018). Thrombospondin receptor $\alpha 2\delta$-1 promotes synaptogenesis and spinogenesis via postsynaptic Rac1. J. Cell Biol. *217*, 3747–3765. https://doi.org/10.1083/JCB.201802057.

51. Danielson, E., Lee, S.H., and Fox, M.A. (2014). SynPAnal: Software for rapid quantification of the density and intensity of protein puncta from fluorescence microscopy images of neurons. PLoS One *9*, e115298. https://doi.org/10.1371/journal.pone.0115298.

52. Schmitz, S.K., Hjorth, J.J.J., Joemai, R.M.S., Wijntjes, R., Eijgenraam, S., de Bruijn, P., Georgiou, C., de Jong, A.P.H., van Ooyen, A., Verhage, M., et al. (2011). Automated analysis of neuronal morphology, synapse number and synaptic recruitment. J. Neurosci. Methods *195*, 185–193. https://doi.org/10.1016/j.jneumeth.2010.12.011.

53. Moreno Manrique, J.F., Voit, P.R., Windsor, K.E., Karla, A.R., Rodriguez, S.R., and Beaudoin, G.M.J. (2021). SynapseJ: An Automated, Synapse Identification Macro for ImageJ. Front. Neural Circ. *15*, 731333. https://doi.org/10.3389/FNCIR.2021.731333.

54. Dzyubenko, E., Rozenberg, A., Hermann, D.M., and Faissner, A. (2016). Colocalization of synapse marker proteins evaluated by STED-microscopy reveals patterns of neuronal synapse distribution in vitro. J. Neurosci. Methods *273*, 149–159. https://doi.org/10.1016/J.JNEUMETH.2016.09.001.

55. Wang, Y., Wang, C., Ranefall, P., Broussard, G.J., Wang, Y., Shi, G., Lyu, B., Wu, C.T., Wang, Y., Tian, L., and Yu, G. (2020). SynQuant: an automatic tool to quantify synapses from microscopy images. Bioinformatics *36*, 1599–1606. https://doi.org/10.1093/BIOINFORMATICS/BTZ760.

56. Irala, D., Wang, S., Sakers, K., Nagendren, L., Ulloa Severino, F.P., Bindu, D.S., Savage, J.T., and Eroglu, C. (2024). Astrocyte-secreted neurocan controls inhibitory synapse formation and function. Neuron *112*, 1657–1675.e10. https://doi.org/10.1016/j.neuron.2024.03.007.

57. Berg, S., Kutra, D., Kroeger, T., Straehle, C.N., Kausler, B.X., Haubold, C., Schiegg, M., Ales, J., Beier, T., Rudy, M., et al. (2019). ilastik: interactive machine learning for (bio)image analysis. Nat. Methods *16*, 1226–1232. https://doi.org/10.1038/s41592-019-0582-9.

58. Eisele, R. (2016). Calculate the intersection area of two circles. https://www.xarg.org/2016/07/calculate-the-intersection-area-of-two-circles/.

59. Irala, D., Wang, S., Sakers, K., Nagendren, L., Ulloa-Severino, F.P., Bindu, D.S., Eroglu, C., and Eroglu, C. (2023). Astrocyte-Secreted Neurocan Controls Inhibitory Synapse Formation and Function 2. Preprint at bioRxiv. https://doi.org/10.1101/2023.04.03.535448.

60. Singh, S.K., Stogsdill, J.A., Pulimood, N.S., Dingsdale, H., Kim, Y.H., Pilaz, L.J., Kim, I.H., Manhaes, A.C., Rodrigues, W.S., Pamukcu, A., et al. (2016). Astrocytes Assemble Thalamocortical Synapses by Bridging NRX1α and NL1 via Hevin. Cell *164*, 183–196. https://doi.org/10.1016/j.cell.2015.11.034.

61. Allen, N.J., Bennett, M.L., Foo, L.C., Wang, G.X., Chakraborty, C., Smith, S.J., and Barres, B.A. (2012). Astrocyte glypicans 4 and 6 promote formation of excitatory synapses via GluA1 AMPA receptors. Nature *486*, 410–414. https://doi.org/10.1038/NATURE11059.

62. Christopherson, K.S., Ullian, E.M., Stokes, C.C.A., Mullowney, C.E., Hell, J.W., Agah, A., Lawler, J., Mosher, D.F., Bornstein, P., and Barres, B.A. (2005). Thrombospondins are astrocyte-secreted proteins that promote CNS synaptogenesis. Cell *120*, 421–433. https://doi.org/10.1016/J.CELL.2004.12.020.

63. Kucukdereli, H., Allen, N.J., Lee, A.T., Feng, A., Ozlu, M.I., Conatser, L.M., Chakraborty, C., Workman, G., Weaver, M., Sage, E., et al. (2011). Control of excitatory CNS synaptogenesis by astrocyte-secreted proteins Hevin and SPARC. Proc. Natl. Acad. Sci. USA *108*, E440–E449. https://doi.org/10.1073/pnas.1104977108.

64. Diniz, L.P., Almeida, J.C., Tortelli, V., Vargas Lopes, C., Setti-Perdigão, P., Stipursky, J., Kahn, S.A., Romão, L.F., De Miranda, J., Alves-Leon, S.V., et al. (2012). Astrocyte-induced synaptogenesis is mediated by transforming growth factor β signaling through modulation of d-serine levels in cerebral cortex neurons. J. Biol. Chem. *287*, 41432–41445. https://doi.org/10.1074/jbc.M112.380824.

65. Eroglu, Ç., Allen, N.J., Susman, M.W., O'Rourke, N.A., Park, C.Y., Özkan, E., Chakraborty, C., Mulinyawe, S.B., Annis, D.S., Huberman, A.D., et al. (2009). Gabapentin Receptor $\alpha 2\delta$-1 Is a Neuronal Thrombospondin Receptor Responsible for Excitatory CNS Synaptogenesis. Cell *139*, 380–392. https://doi.org/10.1016/j.cell.2009.09.025.

STAR★METHODS

KEY RESOURCES TABLE

REAGENT or RESOURCE	SOURCE	IDENTIFIER
**Antibodies**		
AffiniPure Goat anti-Rat IgG + IgM (H + L)	Jackson Immunoresearch	Cat# 112005044 RRID: AB_2338094
AffiniPure Goat anti-Mouse IgG + IgM (H + L)	Jackson Immunoresearch	Cat# 115005044 RRID: AB_2338451
Mouse anti-neural cell adhesion molecule L1 Hybridoma	Developmental Studies Hybridoma Bank	Cat# ASCS4 RRID: AB_528349
Anti-Bassoon antibody	Enzo/Assay Designs	Cat# SAP7F07/VAM-PS003F RRID: AB_2038857
Anti-Gephyrin antibody	Synaptic Systems	Cat# 147002 RRID: AB_2619838)
Anti-Homer1 antibody	Synaptic Systems	Cat# 160002 RRID: AB_2120990
Anti-VGAT antibody	Synaptic Systems	Cat# 131004 RRID: AB_887873
Anti-Vglut1 antibody	Millipore	Cat# AB5905 RRID: AB_2301751
Alexa Fluor 488 goat anti-Mouse IgG (H + L)	Invitrogen	Cat # A11001 RRID: AB_2534069
Alexa Fluor 568 goat anti-Rabbit IgG (H + L)	Invitrogen	Cat# A11011 RRID: AB_143157
Alexa Fluor 647 goat anti-Guinea pig IgG (H + L)	Invitrogen	Cat# A21450 RRID: AB_2535867
Guinea pig anti- VGAT	Synaptic Systems	Cat# 131004 RRID: AB_887873
Rabbit anti-Gephyrin	Synaptic Systems	Cat# 147002 RRID: AB_2619838
Rabbit anti-PSD95	Life Technologies	Cat# 51-6900 RRID: AB_2533914
**Chemicals, peptides, and recombinant proteins**		
2,2,2-tribromoethanol	Sigma	Cat# T48402-25G
2-methyl-2-butanol	Sigma	Cat# 152463-250mL
B27	GIBCO	Cat# 17504044
B27 Plus	GIBCO	Cat# A3582801
BDNF	PeproTech	Cat# 450-02
Boric Acid	Sigma	Cat# B0394
BSA	Sigma	Cat# A4161
BSL1 (Baneiraea Simplicifolia Lectin 1)	Vector Laboratories	Cat# L-1100
CNTF	PeproTech	Cat# 450-13
Cytosine arabinoside (AraC)	Sigma	Cat# C1768
DAPI	Invitrogen	Cat#D1306
DMEM	GIBCO	Cat# 11960
DNaseI	Worthington	Cat# LS002007
DPBS with calcium, magnesium, glucose, and pyruvate	GIBCO	Cat# 14287
DPBS without calcium or magnesium	GIBCO	Cat# 14190144
Fetal Bovine Serum	Thermo Fisher	Cat# 10-437-028

(*Continued on next page*)

Continued		
REAGENT or RESOURCE	SOURCE	IDENTIFIER
Forskolin	Sigma	Cat# F6886
Glycerol	Acros Organics	Cat# 15892-0010
Hydrocortisone	Sigma	Cat# H-0888
Insulin	Sigma	Cat# 11882
L-Glutamine	GIBCO	Cat# 25030-081
Mouse Laminin	Cultrex	Cat# 3400-010-01
N-acetyl cysteine	Sigma	Cat# A8199
n-Propyl gallate	Sigma	Cat# P3130-100G
Neurobasal	GIBCO	Cat# 21103049
Neurobasal minus phenol red	GIBCO	Cat# 12348017
Neurobasal Plus	GIBCO	Cat# A3582901
Normal Goat Serum (NGS)	Thermo Fisher	Cat# 01-6201
Optimal Cutting Temperature solution (OCT)	Tissue Tek	Cat# 4583
Papain	Worthington	Cat# LK003178
Pen/Strep	GIBCO	Cat# 15140
PFA 16%	Electron Microscopy Sciences	Cat# 15710
Poly-D-Lysine	Sigma	Cat# P6407
Sodium Pyruvate	GIBCO	Cat# 11360-070
Tris Base	VWR	Cat# 101174-856
Triton X-100	Roche	Cat# 11332481001
Trypsin Inhibitor	Worthington	Cat# LS003083
Critical commercial assays		
Pierce BCA protein assay kit	Thermo Fisher	Cat# 23225
Deposited data		
Synapse microscopy images	This paper	https://doi.org/10.5281/zenodo.12191805
Experimental models: Cell lines		
Rat primary cortical neurons	This paper	N/A
Rat primary cortical astrocytes	This paper	N/A
Experimental models: Organisms/strains		
Rat: Sprague-Dawley	Charles River	001
Software and algorithms		
SynBot (version 1.1.1)	This paper	Eroglu-Lab/Syn_Bot: Syn_Bot synapse calculation macro for FIJI (github.com) https://doi.org/10.5281/zenodo.12571789
FIJI (version 2.14.0)	NIH	https://fiji.sc/RRID:SCR_002285
Ilastik (version 1.3.3)	Anna Kreshuk's lab (EMBL)	https://www.ilastik.org/ RRID:SCR_015246
Puncta Analyzer (version 2.0)	Ippolito and Eroglu, 2010	https://github.com/toddstavish/puncta-analyzer
R: A Language and Environment for Statistical Computing (version 4.3.3)	R Core Team	https://cran.r-project.org/ RRID:SCR_001905
R package nlme (version 3.1–164)	R Core Team	https://www.rdocumentation.org/packages/nlme/versions/3.1-162 RRID:SCR_015655
Other		
20μm nylon mesh	Elko filtering	Cat# 03–20/14
Vivaspin MWCO 5000; 20 mL tubes	Sartorius	Cat # VS2012
Low protein binding tubes	Eppendorf	Cat# 022431081

EXPERIMENTAL MODEL AND STUDY PARTICIPANT DETAILS

Animals

Mice and rats were used for experiments as specified in the text and figure legends. All mice and rats were used in accordance with the Institutional Animal Care and Use Committee (IACUC) and the Duke Division of Laboratory Animal Resources (DLAR) oversight (IACUC Protocol Numbers A147-17-06 and A117-20-05). All mice and rats were housed under typical day/night conditions of 12-h cycles. For all experiments, age, and sex-matched mice were randomly assigned to experimental groups based on genotypes. The primary rat neurons and astrocytes were isolated from wildtype Crl: CD(SD) Sprague-Dawley rats from Charles River Laboratories (RRID: RGD_734476).

METHOD DETAILS

In vitro synapse assay with astrocyte conditioned media

Cortical tissue digestion

Primary rat astrocytes and neurons were isolated from postnatal day 1 (P1) rat pups. Pups were rapidly decapitated, and brains dissected out and placed into a Petri dish of PBS. Brains were then micro-dissected to isolate the cerebral cortex, and the cortices were chopped into pieces approximately 1 cubic millimeter in volume. A transfer pipet was then cut where it began to taper to create a larger opening. This cut pipet was then used to suck up the cortex chunks, and tissue was transferred to a tube of 7.5 units/mL papain digestion solution (Worthington, Cat# LK003178) by gently tapping on the pipette without depressing it. Cortices were then digested for 45 min at 33°C with a brief swirling at the 15- and 30-min points. The digestion solution is then aspirated off and the tissue is re-suspended in a 2 mg/mL trypsin inhibitor ovomucoid solution (Lo Ovo) (Worthington, Cat# LS003083). Cells were then spun in a centrifuge at 300g for 11 min at room temperature. The supernatant was then removed, and the cells were resuspended in a 4 mg/mL Ovomucoid solution (Hi Ovo). Cells were then spun again at 300g for 11 min and the supernatant removed. The resulting cells were then either used for isolating neurons or astrocytes according to the following sections.

Neuronal isolation by immunopanning

The pelleted cells from the cortical tissue digestion were resuspended in Panning Buffer (0.02% BSA with 0.5 μg/ml insulin in DPBS with calcium, magnesium, glucose, and pyruvate (Thermo Fisher, Cat# 14287080)). The cells were then filtered through a 20μm nylon mesh (Elko Filtering, Cat# 03–20/14) to remove clumps. These filtered cells were then added to a Petri dish which had been treated with Griffonia Simplicifolia Lectin I (Vector Laboratories, Cat# L-1100) for 1 h at room temperature prior to adding the cells. The cells were incubated on this lectin plate for 10 min at room temperature. The plate was then forcefully shaken to resuspend any loosely adhered cells. The solution in the lectin plate was then transferred to a second lectin plate for a 10-min incubation at room temperature. The second lectin plate was then forcefully shaken, and the solution transferred to a plate treated with an anti-rat secondary antibody (Jackson Immunoresearch RRID: AB_2338094) for a 15-min incubation. The anti-rat plate was then shaken, and the solution transferred to a plate treated with an anti-mouse secondary antibody (Jackson Immunoresearch RRID: AB_2338451) for a 15-min incubation. The plate was then shaken, and the solution transferred to a plate treated with an L1CAM antibody (α-L1) (Developmental Studies Hybridoma Bank, RRID: AB_528349) which binds to neurons. The cells were incubated on the α-L1 plate for 45 min at room temperature. The media was then gently removed from the α-L1 plate and the plate was gently washed 5 times with panning buffer. Cells bound to the α-L1 plate were then resuspended in the panning buffer and collected in a conical tube. The cells were then spun for 11 min at 300g. The supernatant was removed, and the cells were resuspended in neuronal growth media (NGM: Neurobasal (Gibco, Cat# 21103049), B27 supplement (Gibco, Cat# 17504044), 2 mM L-Glutamine (Gibco, Cat# 25030-081), 100 U/mL Pen/Strep (Gibco, Cat# 15140), 1 mM sodium pyruvate (Gibco, Cat# 11360-070), 4.2 μg/mL Forskolin (Sigma, Cat# F6886), 50 ng/mL BDNF (PeproTech, Cat# 450-02), and 10 ng/mL CNTF (PeproTech, Cat# 450-13)). The cells were then counted and plated at a low density of 60,000 cells each onto poly-D-lysine-treated (Sigma, Cat# P6407) and mouse laminin (Cultrex, Cat# 3400-010-01) treated glass coverslips in a 24-well plate with neuronal growth media. Cells were then kept in a tissue culture incubator at 37°C with 10% CO_2.

Astrocyte isolation and ACM production

The pelleted cells from the cortical tissue digestion were resuspended in astrocyte growth media (AGM: DMEM (GIBCO, Cat# 11960), 10% FBS (Thermo Fisher, Cat# 10-437-028), 10 mM, hydrocortisone (Sigma, Cat# H-0888), 100 U/mL Pen/Strep, 2 mM L-Glutamine, 5 mg/mL Insulin (Sigma, Cat# 11882), 1 mM Sodium Pyruvate, 5 mg/mL N-acetyl-L-cysteine (Sigma, Cat# A8199)). The cells were then filtered through a 20μm nylon mesh to remove cell clumps. The filtered cells were then spun for 9 min at 300g. The supernatant was then removed, and the cells were resuspended in AGM and counted. 15 million cells were then plated in a T-75 flask with a non-aerating top. Cells were then kept in a tissue culture incubator at 37°C with 10% CO_2 with the cap slightly unscrewed to allow airflow.

On DIV3, astrocyte flasks were washed 3 times with DPBS without calcium or magnesium (Gibco, Cat# 14190144). After the third wash, 20 mL of DPBS without calcium or magnesium was added and the flask was vigorously shaken to remove loosely adherent cells. This shake-off process significantly enriches for astrocytes. Media was then replaced with AGM and flasks were returned to their incubator. On DIV 5, astrocytes were treated with 2.43 mg/mL Cytosine arabinoside (AraC) (Sigma, Cat# C1768) through a full media change. On DIV 7 astrocytes were passaged using 0.05% Trypsin-EDTA. 3 million cells each were then plated into 10 cm culture dishes. On DIV 9, astrocytes were switched to a minimal media (Neurobasal medium minus phenol red (Gibco,

Cat# 12348017), 100 U/mL pen/strep, 2mM L-glutamine, and 1mM sodium pyruvate) for astrocyte conditioning. Astrocytes were then cultured for 5 days without any media change, and on DIV 14, the ACM was collected. The ACM was then centrifuged for 5 min at 1,100g to pellet cellular debris. The supernatant was concentrated by centrifugation for 1 h at 3220 g at 4°C in a 5kDa Cutoff Vivaspin tube (Sartorius, Cat # VS2012). ACM was aliquoted into low protein binding tubes (~100ul per aliquot), flash-frozen in liquid nitrogen, and stored at −80°C until use. An aliquot of each batch of ACM was thawed and used to measure the protein content using the Pierce BCA protein assay kit (Thermo Fisher, Cat# 23225).

Neuron feeding and ACM treatment

On DIV2, neurons were treated with 2.43 mg/mL AraC through a half-media change. A full media change was then performed 24 h later (DIV3) to remove the AraC. Neurons were then given another half-media change on DIV6. Neurons were then fed with ACM on DIV8 and DIV 11 by adding either 50 mg/mL ACM for excitatory synapses or 100 mg/mL ACM for inhibitory synapses in a half media change.

Neuron staining and imaging

On DIV 13, neurons were fixed with 4% paraformaldehyde (Electron Microscopy Sciences, Cat# 15710) in PBS. Cells were then washed 3 times with PBS and then blocked for 30 min at room temperature with antibody blocking buffer (150 mM NaCl, 50 mM Tris-Base, 1% BSA, 100 mM L-lysine 50% normal goat serum (Thermo Fisher, Cat# 01–6201), 0.2% Triton (Roche, Cat# 11332481001). The blocking buffer was then removed, and cells were incubated with primary antibodies against the pre- and post-synaptic markers of interest overnight at 4°C in antibody incubation buffer (150 mM NaCl, 50 mM Tris-Base (VWR, Cat# 101174-856), 1% BSA, 100 mM L-lysine, 10% normal goat serum). The following day cells were washed 3 times with PBS and incubated in fluorescent secondary antibodies in antibody incubation buffer for 2 h at room temperature protected from light. Cells were then washed with PBS 3 times and incubated with 1:10,000 DAPI (Invitrogen, Cat#D1306) for 5 min. Cells were then washed 3 times with PBS and mounted onto coverslips with homemade mounting media (20mM Tris pH 8.0, 90% Glycerol, 0.5% N-propyl gallate) and sealed with nail polish.

Single focal plane images were taken using an inverted Olympus FV3000 confocal laser scanning microscope with a 60X oil immersion objective. The images were taken in a 1024X1024 resolution with a 2X digital zoom. For the picture acquisition, the DAPI channel was used to select neuronal cell bodies distant from other neurons to avoid overlap of cell soma and to do a blind selection of the neurons. DAPI positive nuclei with abnormal morphology were excluded for the analysis. 20 cells for each condition were selected for the analysis. Images were also saved as.bmp extension with only Bassoon (green) and Gephyrin (red channel) for the synaptic analysis.

Mouse brain tissue synapse assay

Mouse perfusion and cryosectioning

P21 α2δ-1 WT and KO mice were euthanized by intraperitoneal injection of 0.6mL of 12.5 mg/mL 2,2,2-tribromoethanol (Avertin) (Sigma, Cat# T48402-25G) followed by exsanguination through transcardially perfusion of tris-buffered saline (TBS: 137mM NaCl, 2.68 mM KCl, 24.8 mM Tris-base (Thermo Fisher, Cat# J75825.A7)). Following TBS perfusion, animals were perfused with warm, 4% paraformaldehyde (PFA) in TBS solution. Brains were then removed and post-fixed in 4% PFA overnight at 4°C. After post-fixation, brains were washed 3 times with TBS and then incubated in 30% sucrose in TBS for 48–72 h. Brains are fully cryopreserved once they sink to the bottom of their container in the 30% sucrose. Brains were then placed in cryomolds and frozen in 50% Optimal Cutting Temperature Compound (OCT) (Tissue Tek, Cat# 4583) in TBS on dry ice. Brains were then kept at −80°C until sectioning. Brains were cryosectioned to a thickness of 15–40 μm and stored in 50% glycerol (Acros Organics, Cat# 15892-0010) in TBS at −20°C.

Tissue section synapse staining and imaging

Three tissue sections per animal were washed 3 times for 10 min with 0.2% Triton X-100 in TBS (TBST) on a shaker at room temperature. Sections were then blocked with 5% normal goat serum in TBST for 1 h at room temperature on a shaker. Sections were then stained with primary antibodies for the pre and postsynaptic markers of interest overnight at 4°C on a shaker. Sections were then washed 3 times in TBST for 10 min and incubated with fluorescent secondary antibodies for 2 h at room temperature on a shaker protected from light. After secondary incubation, sections were washed 3 times with TBST for 10 min. After washing, sections were mounted onto microscope slides and sealed with nail polish.

Stained tissue is ready to image after nail polish is dried and should be imaged within 48 h of secondary staining for most accurate results. Images were acquired using a Leica SP5 confocal microscope with a 63X oil immersion objective. For 1024 X 1024 images, a 1.64X digital zoom was used to achieve an XY resolution of 0.126 μm X 0.126 μm pixel size and a z stack of 15 optical planes was acquired using a 0.34 μm z-step to image ~5 μm of the sample. In general, images were acquired such that confocal settings were optimized on control conditions with the goal of minimizing oversaturated pixels but maintaining real synaptic puncta.

Image analysis

Synapse quantification with Puncta Analyzer

In vitro synapse assay images were analyzed using Puncta Analyer[38] by first converting the images to the RGB format and then running the Puncta Analyzer plugin on each image. A circular ROI was applied to each image with a radius of 301 pixels around the cell body of the imaged neuron. A threshold for each image was chosen to minimize the background while preserving the signal.

In vivo, synapse assay images were Z-projected such that every 3 optical planes were projected to produce 5 max-projections corresponding to 1 μm, per 15-image stack covering the 5 μm. Images were then analyzed with the Puncta Analyzer plugin using default settings. A threshold for each image was chosen by users to minimize the background while preserving the signal.

Synapse quantification with SynBot

Images were analyzed with the SynBot macro using the default settings for the manual thresholding method. A threshold for each image was chosen to minimize the background while preserving the signal. Threshold performance was checked visually using the "_colocs" image SynBot creates that displays which puncta were counted as synapses in each image (see Figures 6 and S2 for example coloc images).

For the ilastik thresholding method, images were first preprocessed into individual color channels using the extract_channels macro (available at https://github.com/Eroglu-Lab/Syn_Bot). These single-channel images were then used to train ilastik projects for each image channel. Each ilastik project was trained using the pixel classification mode in ilastik. A random subset of images from the target color channel was chosen for the training set. For both *in vitro* and *in vivo* synapse analysis, we used all the image features available. We then annotated approximately 20 synapses as label 1 and marked several regions of the background as label 2 on the first image. We then used the live update feature to view the classifications of each pixel and added additional annotations to the first image and subsequent images as necessary. We then saved the ilastik project files and used these for the SynBot ilastik thresholding method (all ilastik project files used for this paper are available at https://github.com/Eroglu-Lab/Syn_Bot).

For the SynQuant thresholding method, images were analyzed using the SynQuant batch thresholding option and SynBot noise reduction. The following SynQuant parameters were used: Simulated data: *Z* score threshold = 10, minimum object size = 10, maximum object size = 100, minimum object fill = 0.5, maximum width to height ratio = 4, z axis multiplier = 1, and estimated noise standard deviation = 12. *In vitro* data: *Z* score threshold = 10, minimum object size = 10, maximum object size = 100, minimum object fill = 0.5, maximum width to height ratio = 4, z axis multiplier = 1, and estimated noise standard deviation = 20. *In vivo* data: *Z* score threshold = 10, minimum object size = 10, maximum object size = 100, minimum object fill = 0.5, maximum width to height ratio = 4, z axis multiplier = 1, and estimated noise standard deviation = 12.

Creation of simulated synapse images

To produce these simulated images, we first used a set of 20 real VGluT1-PSD95 synapse images from Risher et al., 2019. We split the two image channels and then measured the pixel intensity histogram from these images. We generated a Gaussian noise background for each image by multiplying the mean and standard deviation of the original image's histogram by a multiplier (0.00, 0.25, 0.50, 0.75, or 1.00). Since the pixel intensity histogram includes the true signal as well as the background, the 1.00 multiplier will produce an image with a higher background than that of the original image. This makes the range of 0–1 for our noise multiplier represent images with no background at (0.00), moderate background comparable to experimental data (0.25–0.75), and high background beyond what would be acceptable for analysis (1.00). We next took 10 synaptic puncta from each channel of the original image and pasted these onto the background images we generated. Synaptic puncta were pasted into 1000 possible positions such that 334 positions had a red puncta only, 333 positions had a green puncta only, and 333 positions had a red puncta and a green puncta at the same location. Note that the locations for pasting these synaptic puncta were defined by the top left corner of their bounding box. This resulted in varying levels of overlap similar to real synaptic imaging data and explains the precision and recall of SynBot being below 1.0 since many of these synapses were no longer overlapping after being thresholded. The end product of this code was 100 total images with 20 images having each of the 5 background levels. The ImageJ macro code used for generating these simulated images is available at https://github.com/Eroglu-Lab/Syn_Bot).

QUANTIFICATION AND STATISTICAL ANALYSIS

SynBot was used to quantify synapse numbers in the experiments shown. Subsequent statistical analysis was performed with the R statistical software (R Core Team). Student's t-tests were used to test for significance between conditions for the *in vitro* experiments, and linear mixed-effects models were used (R package nlme, R Core Team) to test the significance of *in vivo* experiments and account for the nested structure of the data (multiple z-stacks per image and multiple images per animal). Statistical details of each experiment can be found in the figure legends. For the cell culture experiments, each n represents a neuron that was imaged. For mouse tissue sections experiments, each n represents the average synapse density of a mouse that was included in the experiment. For each mouse, at least 15 max-projection images corresponding to 3 independent z-stacks were used. The full R code for performing the statistical analyses and producing the plots in this paper is available at https://github.com/Eroglu-Lab/Syn_Bot.

Cell Reports Methods, Volume *4*

Supplemental information

SynBot is an open-source image analysis software for automated quantification of synapses

Justin T. Savage, Juan J. Ramirez, W. Christopher Risher, Yizhi Wang, Dolores Irala, and Cagla Eroglu

Table S1 SynBot Functions Defined Related to Figure 2

Step	Function Name	Description
1a	2-Channel Colocalization	The red and green image channels are analyzed for colocalizations.
1b	3-Channel Colocalization	The red, green, and blue image channels are analyzed for triple colocalizations.
1-	Pick Channels	This option allows the user to change the color of the channels in the input images. This is particularly useful since SynBot requires the input images to have the channels to be analyzed in red, green, and blue. SynBot will attempt RGB conversion without this option selected and may have unintended results such as splitting magenta signal across red and blue or analyzing the wrong channels.
2-	Noise Reduction<br>(Recommended)	This option removes background by applying the FIJI Subtract Background function (rolling ball radius = 50) followed by a Gaussian Blur Filter (sigma = 0.57) to aid in object detection.
2-	Brightness Adjustment	This option adjusts the image brightness using the FIJI Enhance Contrast function to have a certain percentage of saturated pixels. This option is not recommended unless images are consistently dim across conditions. Consider adjusting imaging parameters.
3a	Manual	This thresholding method allows the user to manually threshold each channel of each image.
3b	Fixed Value	This thresholding method allows the user to set a single threshold value for each channel that is used for all of the images being analyzed.
3c	Percent Histogram	This thresholding method adjusts the image threshold such that the brightest pixels are kept. The percentage of pixels above threshold for each channel is provided by the user.
3d	FIJI auto	This thresholding method allows the user to apply any of the automated thresholding algorithms in the FIJI auto threshold function.
3e	ilastik	This thresholding method applies a user-trained random forest machine learning model for each channel to threshold the images. Training the model in ilastik is described in Berg et. al.,

		2019 *Nature Methods*. We recommend training the ilastik model on at least 5 images across conditions.
3f	Pre-Thresholded	This thresholding method allows users to input pre-thresholded images into SynBot.
3g	Threshold from File	This thresholding method allows users to enter a csv with threshold values to use for each channel of each image. Importantly, this feature can be used to replicate previous analyses and optimize other analysis parameters while keeping the thresholds the same.
4a	Whole Image	This ROI option analyzes the whole input image.
4b	Auto Cell Body	This ROI option can be used to identify a single cell body automatically and draw a circular ROI around it. This method does not work well if the image includes more than one cell. If this method fails SynBot will prompt the user to use the circle method.
4c	Circle	This ROI option allows the user to select a central point for each image and creates a circle ROI around that point with a set radius.
4d	Cell Territory	This ROI option can be used to automatically identify a cell's territory using a cell fill marker in the blue channel. A mask is created which serves as the ROI for red and green colocalization. This ROI method only works with the 2-Channel colocalization method selected in step 1.
4e	Custom	This ROI allows the user to set a custom ROI for each image.
5 & 6	Red/Green/Blue<br>Min/Max Pixel Size	This field allows the user to set a minimum and maximum pixel area to be counted as a punctum for each channel.
7a	Circular-approximation	This analysis option calculates colocalizations by approximating red and green puncta to circles based on their radii and coordinates and using this to determine if they overlap.
7b	Pixel-overlap	This analysis option uses the FIJI Image Calculator function to identify pixels that contain red and green signal that is above threshold and counts these as colocalizations.
7-	90-degree rotation control	This option rotates the red image 90 degrees to the right and then performs the SynBot analysis as usual. This can be used as a control to test if the number of colocalization observed are greater than what would be seen by chance given the abundance of the red and green markers.

	Experiment Directory	This opens a file explorer dialog to select the image input folder.

Table S2 Circularity of Synaptic Markers Related to Figures 5 and 6

Corresponding Figure	Marker	Average Circularity (Range 0-1) +/- standard deviation
5 E & F	Homer1	0.882 +/- 0.0413
5 E & F	Bassoon	0.897 +/- 0.0162
5 G & H	Gephyrin	0.884 +/- 0.0223
5 G & H	Bassoon	0.911 +/- 0.0124
6 B & C	PSD95	0.922 +/- 0.0282
6 B & C	VGluT1	0.823 +/- 0.0314

A)

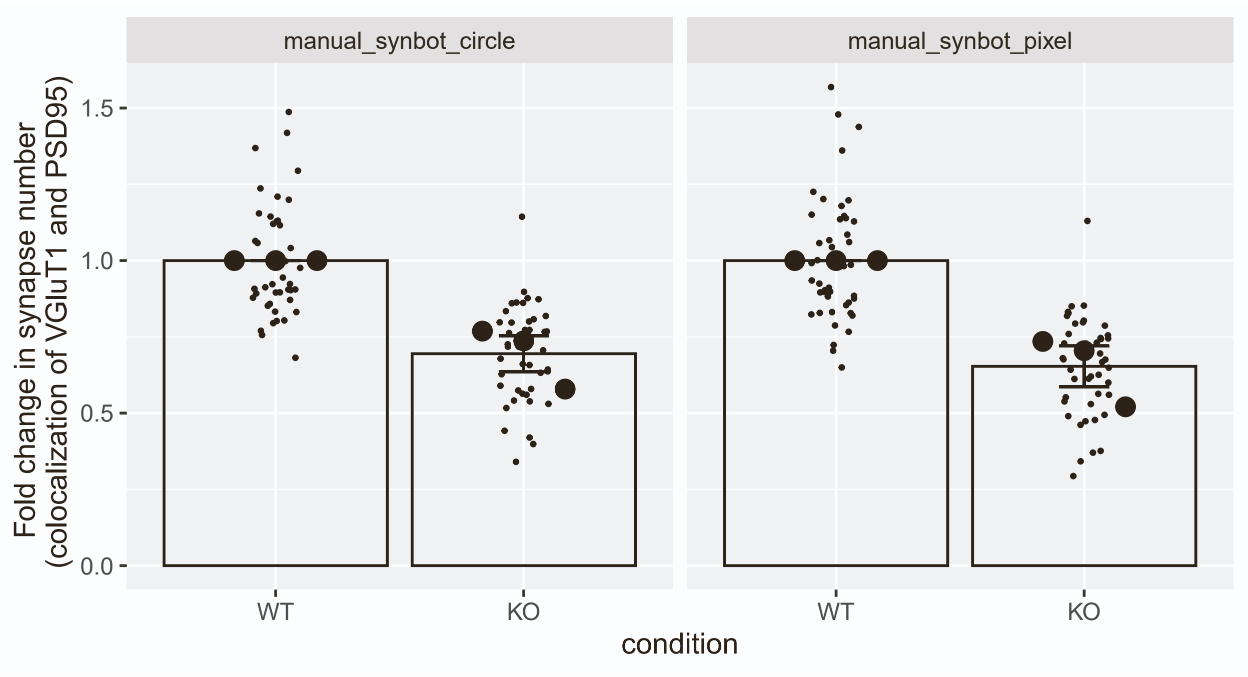
manual_synbot_circle
manual_synbot_pixel
Fold change in synapse number
(colocalization of VGluT1 and PSD95)
1.5
1.0
0.5
0.0
WT
KO
WT
KO
condition

Figure S1 Colocalized Puncta Detected Using Circular Approximation vs. Pixel-by-pixel Analysis Types Related to Figure 6

A) Quantification of VGluT1-PSD95 colocalization using the SynBot manual thresholding method with either the circular approximation or pixel-by-pixel analysis types. Colocalized puncta counts were normalized to the WT average for each experimental pair. Mouse averages are shown as large black dots (N = 3 mice per condition) with individual images shown as small gray dots. Error bars represent 1 standard error of the mean.

Figure S2

Savage et. al., 2024

A)

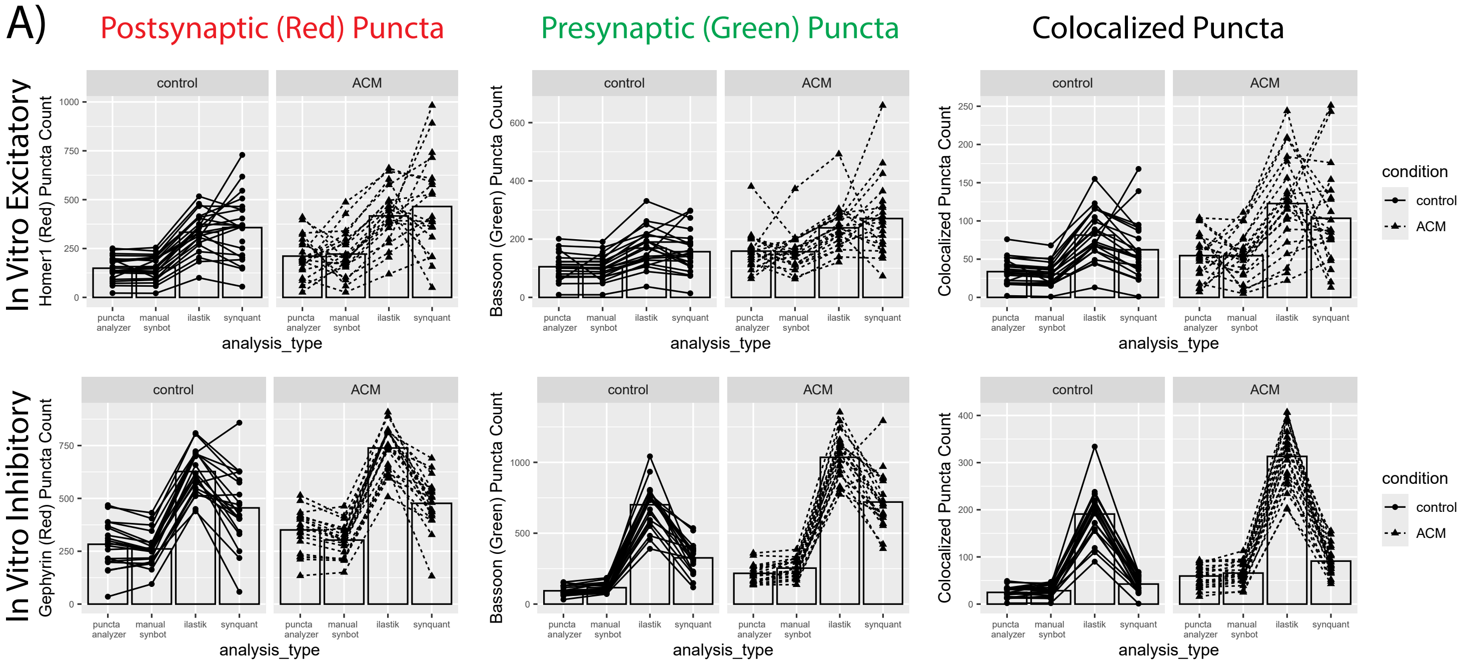

B)

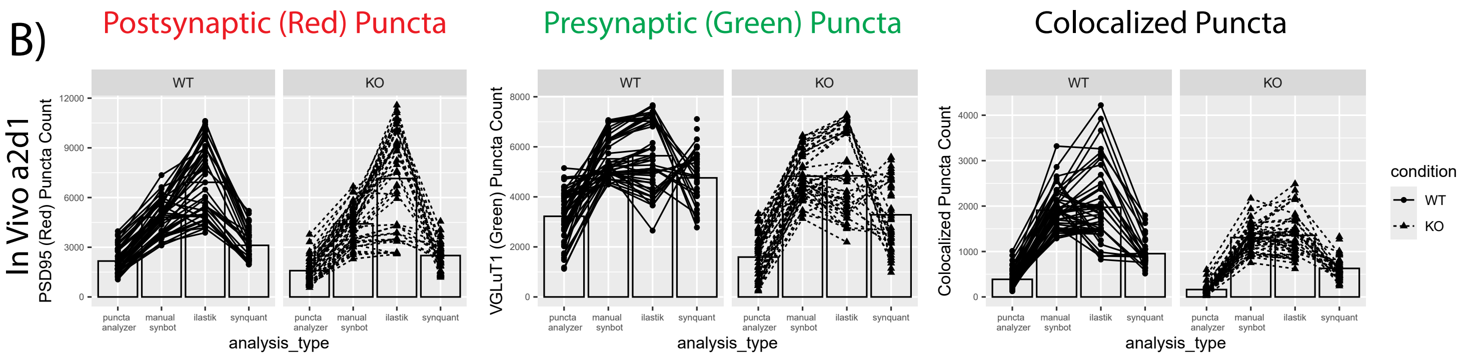

C)

dataset	anova p value	manual_synbot-puncta_analyzer	ilastik-puncta_analyzer	synquant-puncta_analyzer	ilastik-manual_synbot	synquant-manual_synbot	synquant-ilastik
simulated_data_raw	0.916						
invitro_excitatory_normalized	0.896						
invitro_excitatory_raw	4.87E-11	0.998	0	0	0	0	0.151
invitro_inhibitory_normalized	0.138						
invitro_inhibitory_raw	2.00E-16	0.977	0	0.114	0	0.249	0
invivo_excitatory_normalized	0.05	0.096	0.083	0.135	0.999	0.999	0.997
invivo_excitatory_raw	2.00E-16	0	0	0	0.846	0	0

Figure S2 Raw Puncta Counts by Analysis Method Related to Figures 5 and 6

A) Raw postsynaptic (red) puncta, presynaptic (green) puncta, and colocalized puncta counts for *In Vitro* Excitatory and *In Vitro* Inhibitory experiments related to figures 5E and 5G respectively. Lines indicate puncta counts for the same image across analysis methods. Control images are on the left of each plot marked with solid lines and ACM images are on the right side of each plot marked with dashed lines.

B) Raw postsynaptic (red) puncta, presynaptic (green) puncta, and colocalized puncta counts for *In Vivo* a2d1 experiments related to Figure 6C. Lines indicate puncta counts for the same image across analysis methods. WT images are on the left of each plot marked with solid lines and KO images are on the right side of each plot marked with dashed lines.

C) First Column: Two-way ANOVA analysis of data obtained by each experiment shown in figures 4, 5, and 6 testing for the effect of thresholding method on colocalized puncta count. Accompanying columns show pair-wise Tukey's HSD post-hoc tests if the ANOVA was significant.

Figure S3 Savage et. al., 2024

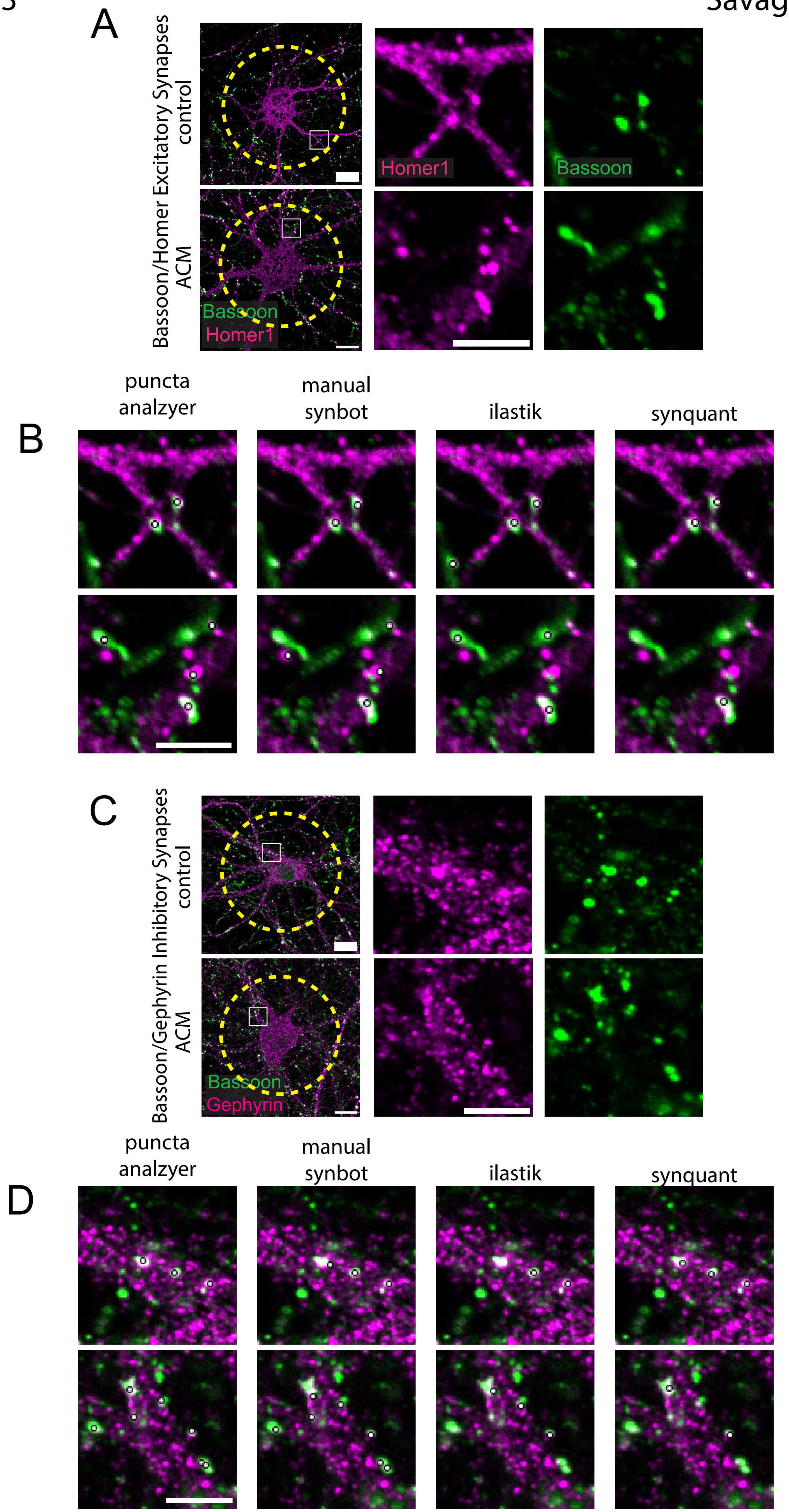

Figure S3 Representative Images of *In Vitro* Synapses Counted by Each Thresholding Method Related to Figure 5

A) Separated Homer1 and Bassoon channels from zoom in excitatory synapse images shown in figure 5E. Scale: 15 μm for full image and 5 μm for zoom in.

B) Zoom in images from figure 5E showing synapses detected by each thresholding method. Small white dots represent the synapses that were detected. Scale: 5 μm.

C) Separated Gephyrin and Bassoon channels from zoom in inhibitory synapse images shown in figure 5G. Scale: 15 μm for full image and 5 μm for zoom in.

D) Zoom in images from figure 5G showing synapses detected by each thresholding method. Small white dots represent the synapses that were detected. Scale: 5 μm.
